# Performance Improvement by Adding Reduced Graphene Oxides in Brush-Painted NiMoO_4_ Nanowires–Polyaniline–Chitosan Composite Flexible Supercapacitor

**DOI:** 10.1021/acs.langmuir.6c01763

**Published:** 2026-06-22

**Authors:** Pei-Yi Yan, Lu-Hong Chen, Li-Da Chiu, Yi-Der Huang, I-Chun Cheng, Jian-Zhang Chen

**Affiliations:** † Graduate School of Advanced Technology, 33561 National Taiwan University, Taipei City 106319, Taiwan; ‡ Institute of Applied Mechanics, National Taiwan University, Taipei City 106319, Taiwan; § Advanced Research Center for Green Materials Science and Technology, National Taiwan University, Taipei City 106319, Taiwan; ∥ Graduate Institute of Photonics and Optoelectronics and Department of Electrical Engineering, National Taiwan University, Taipei City 106319, Taiwan; ⊥ Research Center for Applied Sciences, Academia Sinica, Taipei City 115201, Taiwan

## Abstract

Flexible energy-storage devices are essential for emerging applications such as wearable electronics and soft integrated systems, where mechanical adaptability and long-term stability are required. In this work, a cost-effective brush-painted strategy is developed to fabricate a flexible symmetric supercapacitor based on a NiMoO_4_ nanowire/polyaniline/chitosan/reduced graphene oxide composite electrode. The incorporation of reduced graphene oxide significantly enhances the electrochemical performance by improving the electrical conductivity and interfacial charge transfer, leading to more efficient energy storage. The device operates stably within a voltage window of 0.8 V and delivers an areal capacitance of 125.85 mF/cm^2^ at a low scan rate. In addition, it exhibits excellent cycling durability with 94.5% capacitance retention after 10,000 cycles, as well as robust mechanical flexibility, maintaining stable performance under repeated bending conditions. These results demonstrate that the synergistic design of hybrid electrode materials combined with a simple fabrication approach provides an effective route toward high-performance flexible supercapacitors for next-generation wearable and portable electronics.

## Graphical abstract

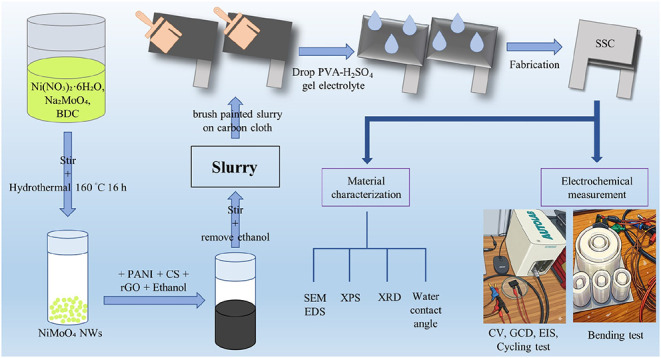

## 1 Introduction

The rapid proliferation of the Internet of Things (IoT), wearable electronics, electronic skins, and foldable displays has exposed the limitations of traditional bulky energy-storage systems, such as cylindrical lithium-ion batteries, which struggle to meet requirements for mechanical flexibility and lightweight integration.^1,2^ Consequently, flexible supercapacitors (FSCs) have emerged as a key research frontier, offering a balance between capacitors and batteries with high power density (>10 kW/kg), rapid charge–discharge rates (seconds), and excellent cycling stability.^3^ Although conventional carbon-based electric double-layer capacitors (EDLCs) deliver high power output, their limited energy density restricts broader applications.^4^ In contrast, transition metal oxides exhibit high theoretical capacitance; however, their practical performance is hindered by low intrinsic electrical conductivity, volume expansion, and sluggish ion diffusion in the bulk phase.^5^

Mixed transition metal oxides (MTMOs), such as NiCo_2_O_4_, ZnCo_2_O_4_, and NiMoO_4_, exhibit superior electrochemical performance compared to single-component oxides due to their enhanced electrical conductivity and multiple accessible oxidation states.^4^ Nevertheless, MTMOs often suffer from limited intrinsic conductivity, structural instability, and particle aggregation during prolonged charge–discharge cycling.^6^ Among them, NiMoO_4_ has attracted considerable attention as a supercapacitor electrode material because of its low cost, natural abundance, environmental benignity, and high theoretical capacitance arising from the synergistic contributions of Ni and Mo.^7^ However, bulk NiMoO_4_ still faces challenges, including poor rate capability and unsatisfactory cycling stability, primarily caused by active material dissolution during repeated cycling.^4^ Engineering NiMoO_4_ into nanostructures effectively addresses these limitations.^8^ In particular, one-dimensional nanowires offer shortened ion diffusion pathways, enhanced electrolyte accessibility, and abundant electroactive sites, thereby facilitating rapid ion/electron transport and improving pseudocapacitive performance.^9^ Therefore, in this work, NiMoO_4_ nanowires (hereinafter referred to as “NiMoO_4_ NWs”) were synthesized via a hydrothermal method and employed as electrode materials to enhance the overall supercapacitor performance. Compared with our previous work (Hsin et al.),^10^ we introduced NiMoO_4_ NWs in this study to incorporate redox-active Ni and Mo centers into a conductive hybrid matrix, aiming to construct an integrated energy-storage network that synergistically combines electric double-layer capacitance (EDLC) with enhanced pseudocapacitance.

Polyaniline (hereinafter referred to as “PANI”) is one of the most promising conductive polymers due to its high pseudocapacitance, facile synthesis, environmental stability, and low cost. Its unique doping/dedoping behavior during redox reactions enables efficient charge storage.^11^ However, PANI suffers from relatively sluggish charge–discharge kinetics compared to porous carbons and poor long-term cycling stability caused by significant volume changes during repeated redox processes, which deteriorate electrode conductivity.^12^ Hybridization with carbon materials, particularly reduced graphene oxide (hereinafter referred to as “rGO”), has been demonstrated to effectively mitigate these limitations. The incorporation of rGO enhances electrical conductivity, buffers volume variation, and provides a high surface area framework for efficient charge transport. Consequently, rGO/PANI nanocomposites exhibit high specific capacitance and improved cycling stability, making them promising candidates for advanced supercapacitor applications.^13^ Moreover, rGO itself possesses a stacked nanoscale architecture with high surface area and excellent conductivity, facilitating rapid electron transport and electrochemical reactions.^14^ In this work, a comparative study was conducted to evaluate the electrochemical performance of devices with and without rGO, aiming to clarify its role in enhancing overall supercapacitor performance.

Chitosan (hereinafter referred to as “CS”) is one of the most important biopolymers. In recent years, CS has been successfully applied in the fabrication of various supercapacitors. Among all polysaccharides, CS possesses several significant advantages, including biocompatibility, biodegradability, nontoxicity, excellent chemical resistance, and favorable electrochemical properties.^11^ However, CS is inherently nonconductive. Adding specific nanoparticles can improve the bioadhesive characteristics of CS since metal oxides are particularly effective at promoting fast electron transfer between the electrode and the electrolyte.^15^ In this study, NiMoO_4_ NWs were employed to provide abundant redox-active sites, while their one-dimensional architecture is expected to facilitate ion transport kinetics. PANI contributes additional pseudocapacitance through its reversible doping/dedoping process and simultaneously forms a conductive network that enhances interfacial charge transfer. rGO serves as a highly conductive scaffold, promoting rapid electron transport, increasing the effective surface area, and buffering structural deformation during cycling. CS, as a biopolymeric binder and structural stabilizer, improves the mechanical integrity between active components as well as their adhesion to the substrate.

In terms of fabrication, the materials were deposited onto the substrate by using a cost-effective brush-painted method. Compared with conventional techniques such as chemical vapor deposition and magnetron sputtering, the brush-painting method offers distinct advantages, including facile operation under ambient conditions and versatile electrode deposition. Moreover, they are not constrained by equipment complexity, ink formulation, or substrate type, making them a highly flexible and scalable fabrication strategy.^16^ Table S1 summarizes representative fabrication methods for supercapacitor electrodes, including their cost, substrate compatibility, scalability, and key features.

Regarding the substrate, carbon cloth (hereinafter referred to as “CC”) was selected as the flexible current collector. CC has been widely employed as a substrate for supercapacitor electrodes due to its excellent flexibility, chemical stability, corrosion resistance, and efficient ion diffusion pathways. Compared with metallic mesh current collectors, it represents a more sustainable and cost-effective alternative. In addition, it exhibits higher resistance to oxidation than nickel foam, contributing to improved long-term durability.^17^ Structurally, CC possesses a three-dimensional interconnected porous network that provides a large accessible surface area. Its woven architecture further enhances mechanical robustness and ensures long-term structural stability during repeated electrochemical cycling.^18^

Beyond material and fabrication considerations, device architecture also plays a critical role in determining overall electrochemical performance. Although asymmetric supercapacitors (hereinafter referred to as “ASCs”) are widely adopted to enlarge the operating voltage window and enhance energy density, symmetric supercapacitors (hereinafter referred to as “SSCs”) offer several intrinsic advantages.^19^ Owing to the use of identical electrode materials, SSCs exhibit more balanced charge–discharge behavior, simplified device fabrication, and reduced issues related to capacitance mismatch and potential imbalance. Moreover, symmetric configurations often demonstrate superior cycling stability and mechanical reliability, particularly in flexible or wearable devices.^20^ Therefore, despite the energy density advantage of ASCs, symmetric architectures remain attractive for applications requiring structural simplicity and long-term stability. In this study, a symmetric configuration was adopted to achieve enhanced structural stability and long-term electrochemical durability.

## 2 Experiment

### 2.1 Preparation of NiMoO_4_ NWs

NiMoO_4_ NWs were synthesized via a hydrothermal procedure. The hydrothermal precursor was formed by mixing nickel­(II) nitrate hexahydrate (Ni­(NO_3_)_2_·6H_2_O, 98%), sodium molybdate (Na_2_MoO_4_, 99%), and 1,4-benzenedicarboxylic acid (BDC, 99%; all supplied by Thermo Scientific) in 160 mL of deionized water. The mixture was magnetically stirred at 300 rpm for 1 h at room temperature until a clear solution was obtained.^21^

The prepared solution was then placed into a Teflon-lined stainless steel autoclave, tightly sealed, and maintained at 160 °C for 16 h. After naturally cooling to room temperature, the obtained precipitate was collected in a beaker, and the supernatant was decanted. The collected product was then dried at 50 °C for 6 h. Finally, the dried powder was ground using a mortar and pestle to obtain fine, dry NiMoO_4_ powder (NiMoO_4_ NWs) with reduced particle agglomeration.

### 2.2 Preparation of CS Solution

To prepare the CS solution, 0.3 g of chitosan powder (sourced from shrimp shells, degree of deacetylation ≥75%, Sigma-Aldrich) was dissolved in 20 mL of 0.1 M acetic acid (purity >99.5%, AUECC). The mixture was then heated in a water bath using a magnetic stirrer at 50 °C and stirred continuously at 300 rpm for 2 h. After the CS powder was completely dissolved, heating was stopped, and the solution was allowed to cool while stirring at 300 rpm for an additional hour. The resulting solution was used as the CS solution.

### 2.3 Preparation of NiMoO_4_ NWs/PANI/CS/rGO Slurry

The slurry was prepared by mixing 0.05 g of NiMoO_4_ NWs, 0.05 g of PANI particles (average molecular weight >15,000, powder, infusible), 0.05 g of rGO (purity: 99%, thickness <5 nm, sheet diameter: 0.1–5 μm, Golden Innovation Business Co., Ltd.), and 3.625 g of CS solution. Subsequently, 1.5 g of ethanol was added as a solvent, and the mixture was continuously stirred at 300 rpm at ambient temperature for 48 h. Afterward, ethanol was removed from the mixture using a rotary evaporator at 55 °C for 150 s, yielding the NiMoO_4_ NWs/PANI/CS/rGO slurry.

### 2.4 Fabrication of Electrode

The slurry was applied onto carbon cloth with an area of 2 × 3 cm^2^ by a brush-painted method. The painting process was repeated three times in a back-and-forth manner to ensure uniform coverage of the slurry on the carbon cloth surface. Subsequently, the coated electrodes were put in an oven and dried at 100 °C for 10 min to obtain a slurry-coated carbon cloth electrode. This procedure was repeated once to prepare two electrodes for subsequent experiments.

### 2.5 Preparation of Gel Electrolyte

1.5 g of poly­(vinyl alcohol) (PVA; 99 + % hydrolyzed, Aldrich) was mixed with 15 mL of 1 M H_2_SO_4_ solution. The mixture was heated in a water bath at 80 °C and magnetically stirred at 200 rpm until a clear solution without any visible precipitates was obtained. Subsequently, the heating was turned off while stirring was continued until the solution cooled to room temperature, yielding the PVA–H_2_SO_4_ gel electrolyte.

### 2.6 Fabrication of Flexible SSC

Initially, a 1 M PVA–H_2_SO_4_ electrolyte was applied onto both electrode surfaces, creating a thin layer on each. The electrodes were subsequently allowed to dry at ambient temperature. To achieve sufficient electrolyte thickness, this drop-coating procedure was repeated two additional times, resulting in a total of three layers. Prior to device assembly, an extra layer of PVA–H_2_SO_4_ electrolyte was deposited on each electrode. The two electrodes were then aligned to construct a sandwich-type configuration. Gentle pressure was applied to both sides to guarantee complete contact between the electrodes and the gel electrolyte. Following assembly, the device was allowed to dry naturally at room temperature for 24 h. This process yielded an assembled flexible symmetric supercapacitor (SSC), and the device is referred to as “NiMoO_4_ NWs/PANI/CS/rGO@CC.” A schematic illustration of the SSC fabrication procedure is shown in Figure 1.

**Figure 1:**
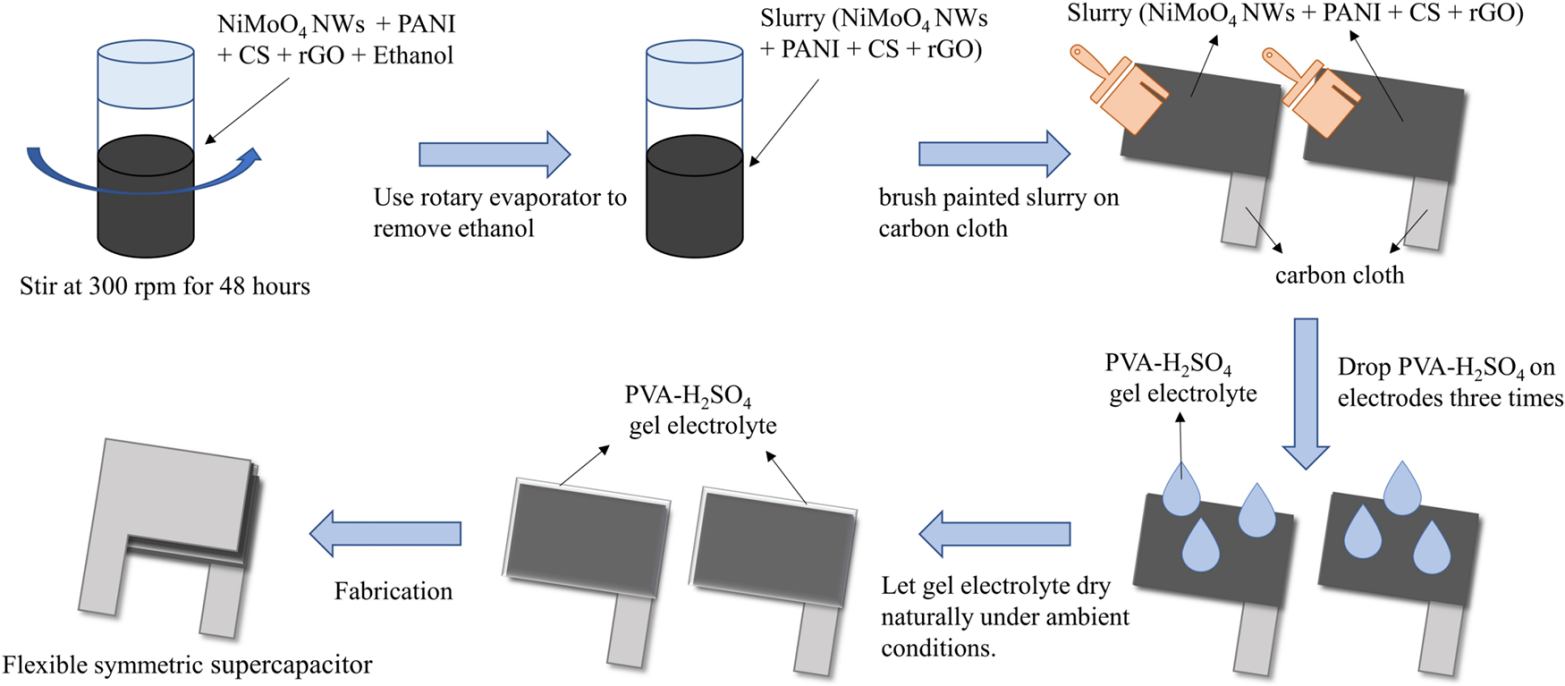
Procedure for fabricating the flexible symmetric supercapacitor (SSC).

### 2.7 Characterization Methods

The surface morphology and crystal structure of NiMoO_4_ NWs/PANI/CS/rGO@CC were examined using scanning electron microscopy (SEM; JSM-7800F Prime, JEOL, Tokyo, Japan) and X-ray diffraction (XRD; D8 Advance Plus 4, Bruker). The wettability of the slurry-coated carbon cloth was assessed via water contact angle measurements (Sindatek, model 100SB, Taipei, Taiwan). Additionally, the chemical composition and surface bonding states of the electrodes were analyzed by X-ray photoelectron spectroscopy (XPS; Sigma Probe, Thermo VG Scientific).

The electrochemical performance of the SSC was evaluated using an electrochemical workstation (Autolab, PGSTAT204, Metrohm, Utrecht, The Netherlands), including cyclic voltammetry (CV), galvanostatic charge–discharge (GCD), and electrochemical impedance spectroscopy (EIS, 0.1–100,000 Hz). Long-term cycling stability was measured by a battery testing system (3C Digital Cell Testing System, Neware, CT-4008Tn-5 V6A-S1-FU). In addition, bending tests were conducted to demonstrate the feasibility of carbon cloth as a flexible substrate.

## 3 Results and Discussion

### 3.1 Material Characterization

Figure 2 shows the SEM images of the pure carbon cloth (CC) and the NiMoO_4_ NWs/PANI/CS/rGO slurry brush-painted on CC at various magnifications. As shown in Figure 2(a), the bare CC exhibits a typical interwoven fiber structure, forming a three-dimensional conductive network. This microstructure allows full infiltration of the gel electrolyte. As shown in Figure 2(b,c), after brush-painting with the NiMoO_4_ NWs/PANI/CS/rGO slurry, the material was successfully adhered to the carbon cloth fibers, and the interfiber voids were partially filled, significantly increasing the effective surface area. At higher magnifications (Figure 2(d–f)), the NiMoO_4_ NWs are uniformly distributed and interwoven with the polymer matrix, indicating that the presence of PANI and CS facilitates the adhesion between the materials and the substrate.^22^ This roughened, hierarchical structure is expected to provide abundant electroactive sites and shorten ion diffusion pathways, thereby enhancing the electrochemical performance.

**Figure 2:**
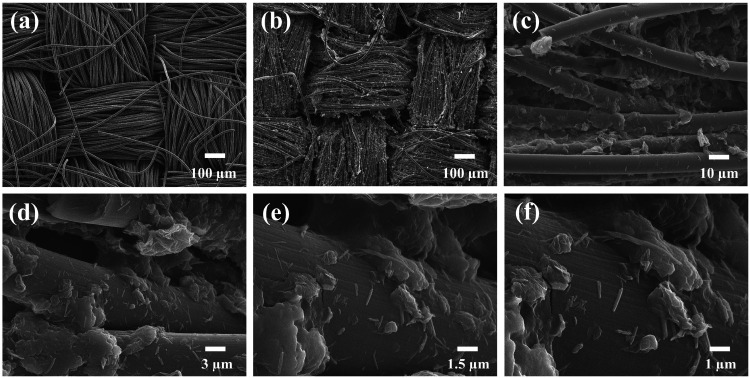
SEM images of (a) pure carbon cloth (CC) at 100× magnification; (b–f) NiMoO_4_ NWs/PANI/CS/rGO@CC at different magnifications (100×, 1000×, 3000×, 7000×, and 10,000×).

Figure 3 shows the EDS elemental mapping of the NiMoO_4_ NWs/PANI/CS/rGO slurry brush-painted on CC at a magnification of 3000×. Figure S1 and Table S2 present the corresponding EDS spectra and atomic ratio contents, respectively. The strong C signal originates mainly from the CC substrate and the PANI/CS/rGO in the slurry, with a high content of 85.10%. The O signal is associated with the metal oxide component and oxygen-containing functional groups. The N element is attributed to the conductive polymer PANI and CS. In addition, although the contents of Ni and Mo are relatively low, they are clearly observed and uniformly distributed on the electrode surface, further indicating the successful synthesis of NiMoO_4_ NWs. Besides EDS, XPS analysis was performed to investigate the distribution of chemical composition on the electrode surface.

**Figure 3:**
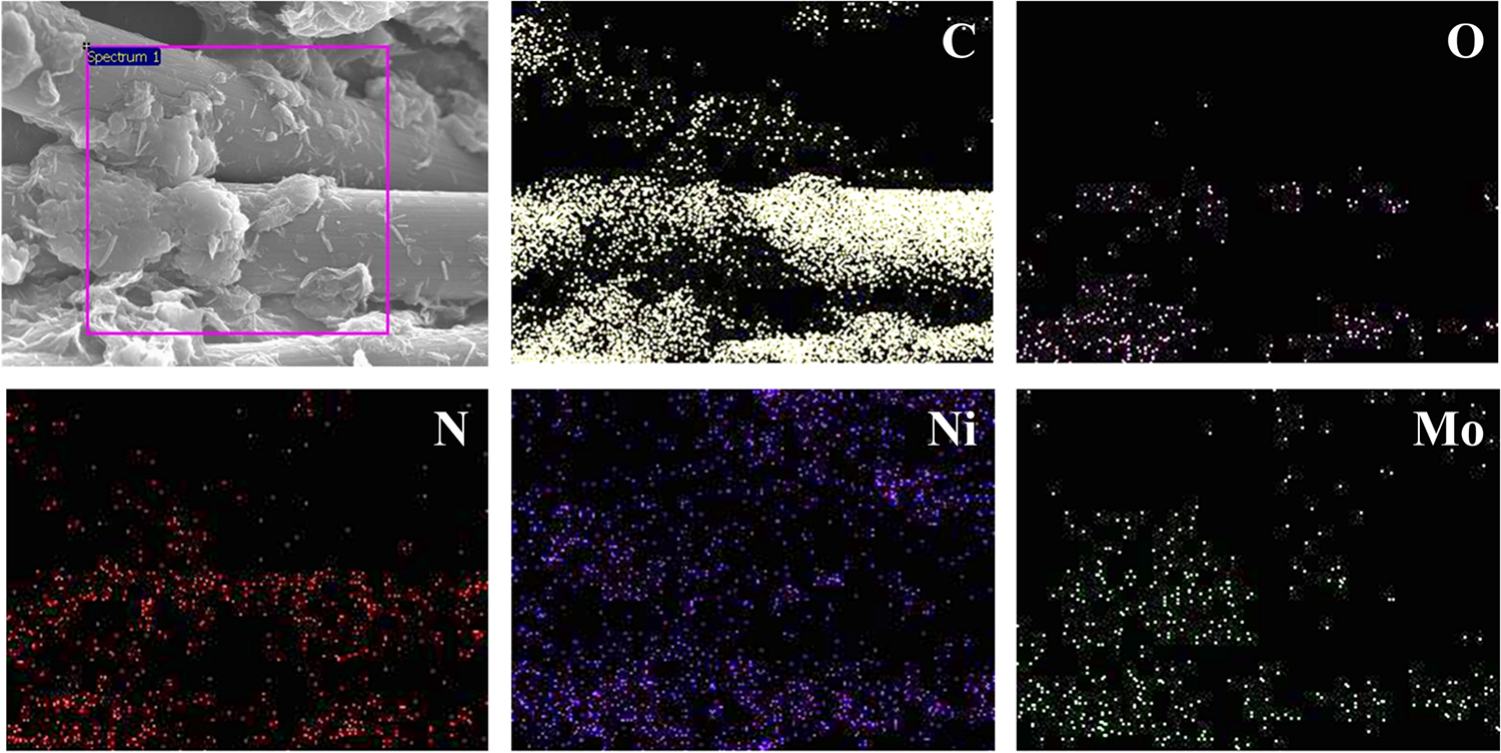
EDS images of NiMoO_4_ NWs/PANI/CS/rGO@CC with 3000 × magnification.

Figure 4 shows the XPS analysis of NiMoO_4_ NWs/PANI/CS/rGO@CC, aimed at investigating the chemical bonding states and oxidation states atop the electrode surface. Figure 4(a) shows the survey spectrum, revealing that the primary elements in the slurry include C 1s, O 1s, N 1s, Ni 2p, and Mo 3d. From comparison with Table S2 and the surface atomic ratios obtained from XPS analysis (Table S3), carbon is the most abundant element in both the surface and subsurface of the electrode, while the oxygen content is significantly higher at the electrode surface than in the subsurface. In contrast, the contents of Ni and Mo showed no obvious variation.

**Figure 4:**
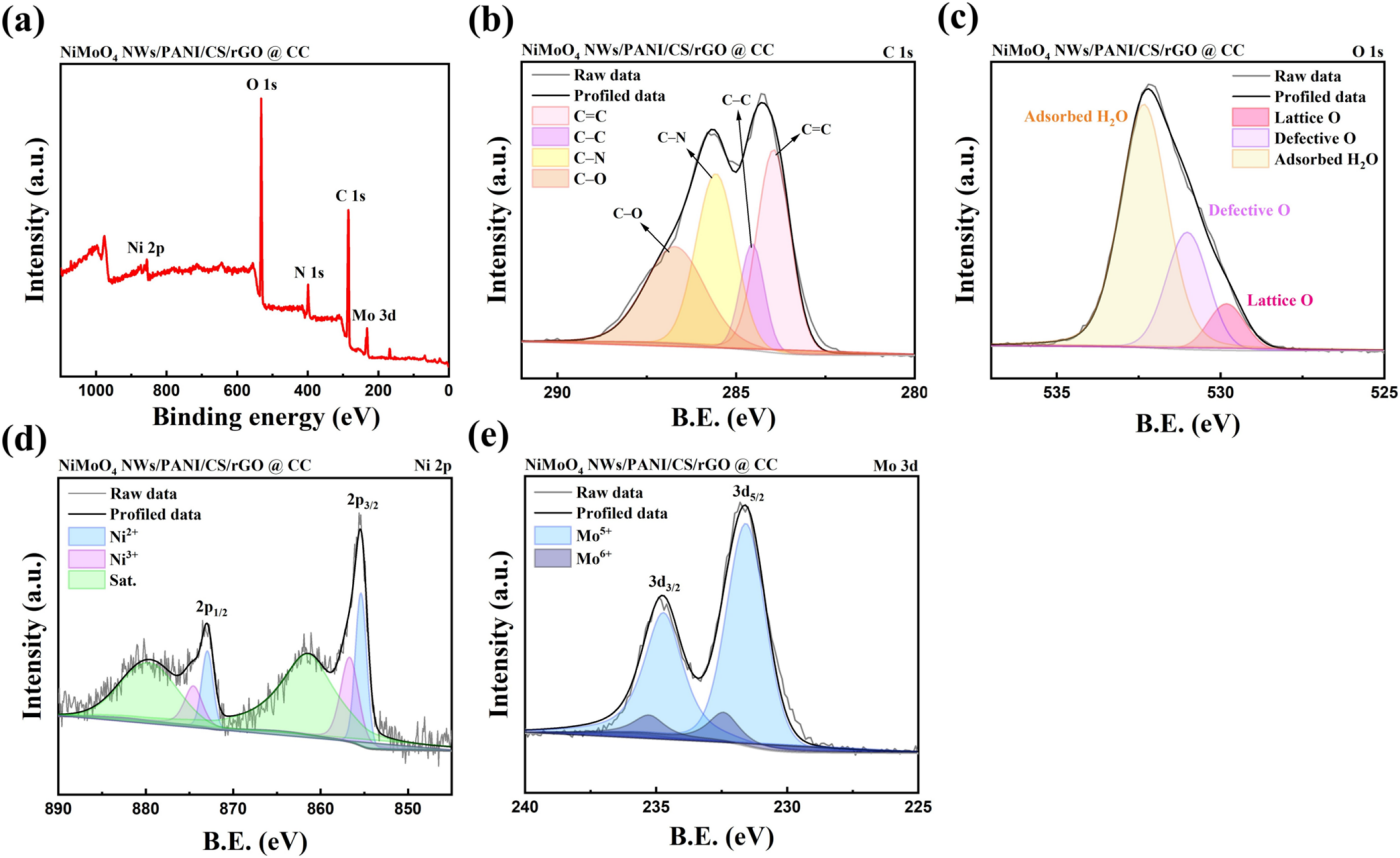
XPS survey spectra of NiMoO_4_ NWs/PANI/CS/rGO @ CC. (a) Full survey spectra; high-resolution XPS (HRXPS) spectra of (b) C 1s, (c) O 1s, (d) Ni 2p, and (e) Mo 3d.

Figure 4(b) and Table 1 show the C 1s high-resolution XPS (HRXPS) spectrum and the corresponding component ratios of different bonding configurations. Four types of carbon bonds are identified: C=C, C–C, C–N, and C–O, with binding energies of 283.86, 284.52, 285.7, and 286.6 eV, respectively.^10,23^ The C=C and C–C bonds primarily arise from the carbon cloth and rGO, while the C–N and C–O bonds are mainly associated with the functional groups of PANI and CS. The C–O bonds may also arise from interactions between the materials and environmental oxygen. It has been reported that an appropriate amount of functional groups, such as C–N and C–O, can enhance the performance of supercapacitors by contributing to pseudocapacitance and improving the wettability between the electrode and electrolyte.^24^

**Table 1:** Component Ratios of C Obtained from XPS Analysis of C 1s Spectra

component ratio of C 1s				
sample	C=C (%)	C–C (%)	C–N (%)	C–O (%)
NiMoO_4_ NWs/PANI/CS/rGO@CC	30.82	11.17	30.10	27.91

As shown in Figure 4(c), the HRXPS of O 1s can be deconvoluted into three distinct components, corresponding to lattice oxygen (lattice O), defect oxygen (defective O), and surface-adsorbed oxygen species (adsorbed H_2_O), respectively. The corresponding binding energies are located at 529.8, 531.0, and 532.3 eV, respectively.^21^ Table 2 lists the component ratios of Lattice O, Defective O, and Adsorbed H_2_O. The high adsorbed H_2_O content can be attributed to the synergistic effects of the high specific surface area of NiMoO_4_ NWs, the abundant polar functional groups (amino and hydroxyl) provided by CS,^25^ and the defect sites in rGO, which facilitate the adsorption of water molecules and oxygen species from the environment.^26^ The second most abundant species is defective O, indicating the presence of partial oxygen vacancies. These oxygen vacancies can provide additional active sites for surface redox reactions, thereby enhancing the electrochemical performance.^27^

**Table 2:** Component Ratios of O Obtained from XPS Analysis of O 1s Spectra

component ratio of O 1s			
sample	lattice O (%)	defective O (%)	adsorbed H_2_O (%)
NiMoO_4_ NWs/PANI/CS/rGO@CC	8.72	25.27	66.01

Figure S2 shows the N 1s HRXPS spectrum of the NiMoO_4_ NWs/PANI/CS/rGO @CC. The N 1s spectrum can be deconvoluted into three distinct peaks, corresponding to pyridinic-N, pyrrolic-N, and graphitic-N, with binding energies of 398.3, 399.9, and 400.9 eV, respectively.^28^ It is believed that these nitrogen-containing functional groups improve the electrode’s electrocatalytic activity.^29^ Furthermore, earlier research has shown that nitrogen doping into carbon-based materials can enhance electronic conductivity and optimize surface reactivity, thereby improving their electrochemical properties.^30^ Therefore, we anticipate that the synergistic interaction among PANI, CS, and rGO would effectively enhance the capacitive performance of the electrode. This expectation is further confirmed by the subsequent electrochemical measurements.

As shown in Figure 4(d), the Ni 2p HRXPS spectrum exhibits characteristic peaks at binding energies of 855.1 and 872.5 eV, which correspond to the Ni^2+^ 2p_3_/_2_ and Ni^2+^ 2p_1_/_2_ electronic states, respectively. The peaks located at 856.7 and 874.5 eV are assigned to the Ni^3+^.^31,32^ The green peaks in the figure were identified as satellite peaks. The analysis results indicate the coexistence of Ni^2+^ and Ni^3+^ oxidation states. As shown in Figure 4(e), the HRXPS spectrum of Mo 3d exhibits peaks at 232.4 and 235.5 eV, which are assigned to the Mo^6+^ 3d_5_/_2_ and 3d_3_/_2_ states, respectively. The peaks at 231.5 and 234.6 eV correspond to Mo^5+^ species.^21,33^ The predominance of Mo^5+^ suggests the possible occurrence of partial reduction processes, which may be further supported by the O 1s spectrum (Figure 4(c)). The pronounced peak associated with defective oxygen indicates the presence of oxygen vacancies. These oxygen vacancies are considered to act as electron donors and may contribute to the partial reduction of Mo, as well as to the improved pseudocapacitive performance of the NiMoO_4_ NWs/PANI/CS/rGO composite.^34^ Overall, the coexistence of mixed-valence states, oxygen vacancies, and heteroatom-containing functional groups is expected to synergistically contribute to improved electrochemical performance by facilitating electronic conductivity, accelerating charge-transfer kinetics, and enhancing electrolyte accessibility within the electrode structure.

Figure 5(a) shows the water contact angle measurement of the NiMoO_4_ NWs/PANI/CS/rGO@CC electrode to evaluate its surface wettability. For comparison, Figure S3 shows the water contact angle of the NiMoO_4_ NW/PANI/CS@CC electrode, which exhibits highly hydrophilic behavior. Such strong hydrophilicity facilitates rapid electrolyte infiltration, thereby enhancing ion diffusion and charge storage capability.^35^ As demonstrated in Figure 5(a), the NiMoO_4_ NWs/PANI/CS/rGO@CC electrode displays a water contact angle of 96.72°, showing a relatively hydrophobic surface. The enhanced water repellency of the electrode is ascribed to the existence of rGO. The water-repellent character of rGO originates from the elimination of polar oxygen-containing groups and the reestablishment of its nonpolar sp^2^ carbon framework.^36^ However, a moderate water contact (96.72°) angle helps enhance the structural stability of the electrode, thereby improving capacitance retention.^37^ In this study, although the incorporation of rGO increases the surface hydrophobicity, it still exerts a positive effect on the overall electrochemical performance. Furthermore, adding rGO increases the active materials for EDLC. This also improves the areal capacitance.

**Figure 5:**
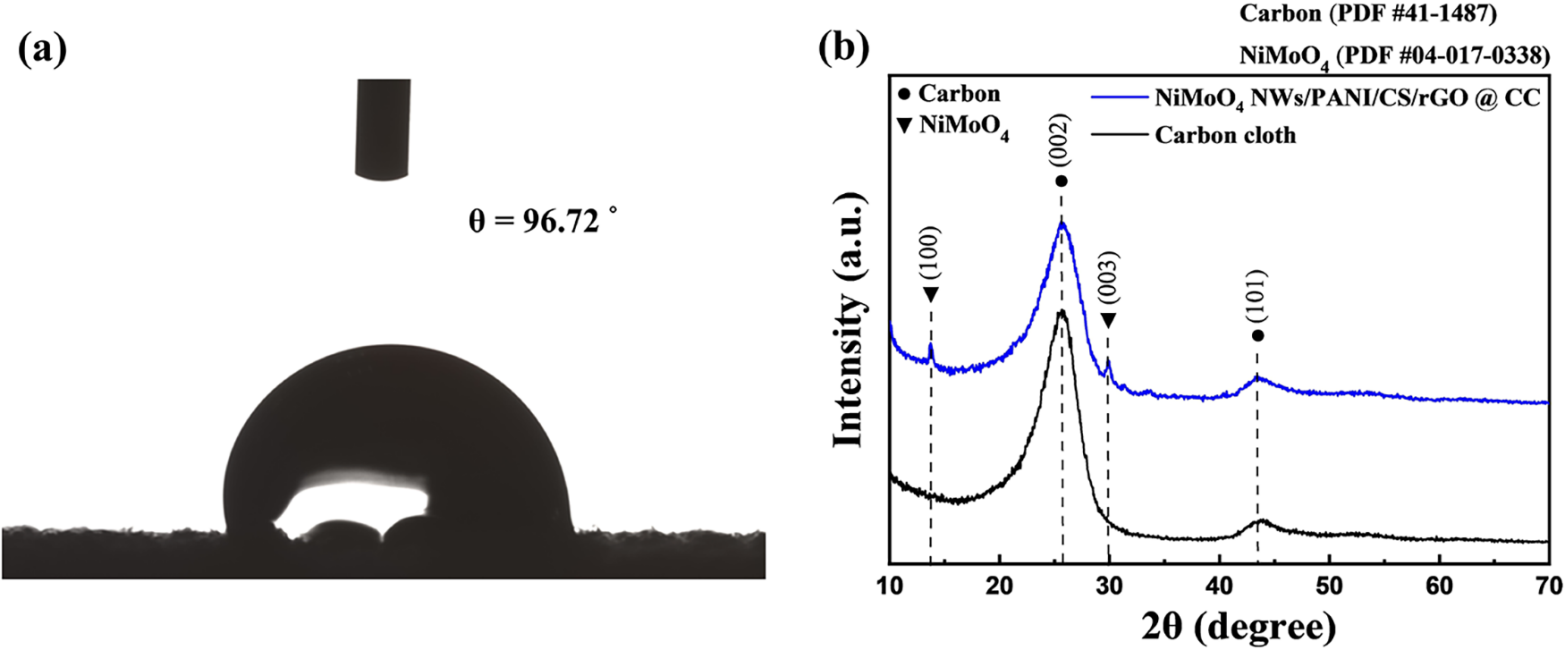
(a) Water contact angle and (b) XRD patterns of NiMoO_4_ NWs/PANI/CS/rGO@CC.

Figure 5(b) shows the X-ray diffraction (XRD) patterns of NiMoO_4_ NWs/PANI/CS/rGO@CC and pure carbon cloth, highlighting their crystalline structural characteristics. For the carbon cloth, two characteristic diffraction peaks located at approximately 26° and 43° are observed, corresponding to the (002) and (101) planes of the carbon substrate (PDF #41–1487), respectively.^38^ After the slurry was brush-painted onto the substrate, additional diffraction peaks emerged at around 13.57° and 29.78°, which indexed to the (100) and (003) crystal planes of triclinic (anorthic) NiMoO_4_ (PDF #04–017–0338),^21^ confirming the successful deposition of NiMoO_4_ NWs on the carbon cloth. No other distinct diffraction peaks are detected, which can be attributed to the low intrinsic crystallinity of the other components. Previous studies have reported that PANI typically exhibits low crystallinity and predominantly amorphous characteristics,^39^ while CS and rGO generally display a broad and weak peak in their XRD patterns, indicating their amorphous characteristics.^40,41^ Amorphous materials, owing to their unique porous structures, excellent cycling stability, and rapid charge–discharge capability, are considered among the most promising active materials for high-performance supercapacitors.^42^

### 3.2 Electrochemical Measurement

The electrochemical performance of the devices was compared with and without the incorporation of rGO, aiming to investigate whether the addition of carbon materials into the slurry could enhance the performance of the supercapacitors. In this study, two identical electrodes were assembled to form SSC, and the device was evaluated using a two-electrode system. Areal capacitance (*C*_A_) was obtained according to the below equation

$$C_{A}=\frac{1}{\Delta V\times v\times A}\int_{V_{A}}^{V_{C}}I\left(V\right)dV$$

Here, $\int_{V_{A}}^{V_{C}}I\left(V\right)dV$ represents the total area enclosed by the CV curve, Δ*V* is the potential window, *v* is the scan rate, and *A* is the electrode’s geometric surface area.^43^ In cyclic voltammetry (CV), the current response induced by potential variation is recorded, and the resulting CV curves can be used to identify the type of electrochemical processes. Specifically, CV analysis can distinguish between electrical double-layer capacitors (EDLC), pseudocapacitors (PC), and battery-type behaviors. The CV profile of an EDLC typically exhibits a nearly rectangular shape, whereas most pseudocapacitive materials display reversible redox peaks superimposed on the capacitive curves. Moreover, CV allows the investigation of the potential window and the reversibility of anodic and cathodic processes.^44^ The primary energy-storage mechanism of EDLC involves the formation of an electric double layer at the electrode–electrolyte interface, while PC stores energy through Faradaic redox reactions, which may occur with or without ion intercalation.^45^ Figure 6 shows the CV analysis conducted at scan rates of 200, 20, and 2 mV/s. At a high scan rate of 200 mV/s, the CV curve exhibits a highly symmetric quasi-rectangular shape, suggesting a dominant EDLC behavior with partial PC contribution. As the scan rate decreases, distinct redox peaks become more pronounced. A lower scan rate allows ions more time to penetrate and interact with the electrode surface, thereby enhancing the PC effect and resulting in higher areal capacitance. Conversely, at high scan rates, the ions have insufficient time to undergo redox reactions, reducing the contribution of PC and leading to lower capacitance values.^46^ Notably, at all scan rates, the integrated area of the CV curves for NiMoO_4_ NWs/PANI/CS/rGO@CC is significantly larger than the sample without rGO. Specifically, at 200 mV/s, the peak current of the NiMoO_4_ NWs/PANI/CS/rGO@CC electrode reaches approximately 50 mA, nearly ten times higher than that of the electrode without rGO. As shown in Table 3, the areal capacitance values of the two electrodes differ markedly, particularly at 20 and 2 mV/s, where NiMoO_4_ NWs/PANI/CS/rGO@CC exhibits areal capacitances of 99.86 and 125.85 mF/cm^2^, respectively; these values are more than ten times higher than the rGO-free counterpart. These results indicate that the incorporation of rGO effectively enhances the electrical conductivity of the material.

**Figure 6:**
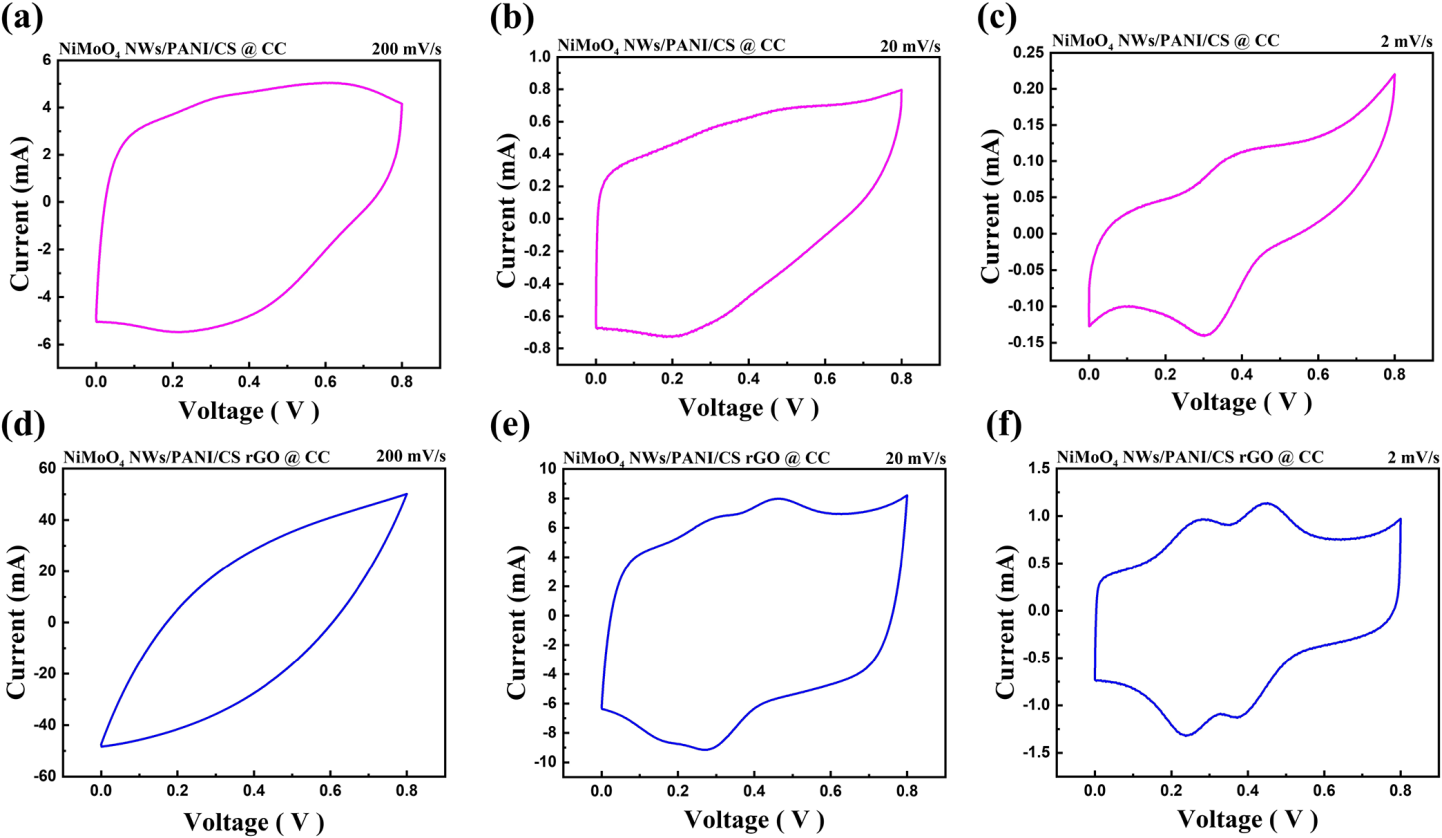
CV curves of NiMoO_4_ NWs/PANI/CS@CC at different scan rates of (a) 200 mV/s, (b) 20 mV/s, and (c) 2 mV/s; CV curves of NiMoO_4_ NWs/PANI/CS/rGO@CC at different scan rates of (d) 200 mV/s, (e) 20 mV/s, and (f) 2 mV/s within a potential window of 0–0.8 V.

**Table 3:** Areal Capacitance of NiMoO_4_ NWs/PANI/CS@CC and NiMoO_4_ NWs/PANI/CS/rGO@CC Calculated from the CV Curves in Fig. 6

areal capacitance (mF/cm^2^)			
	potential scan rate (mV/s)		
sample	200	20	2
NiMoO_4_ NWs/PANI/CS@CC	6.32	7.83	11.59
NiMoO_4_ NWs/PANI/CS/rGO@CC	32.58	99.86	125.85

The charge storage behavior of the electrode can be investigated through Dunn’s method, which is generally calculated by the equation

$$i=av^{b}$$

log(i)=log(a)+blog(v)

where *i* is the peak current, *v* is the scan rate, and *a* and *b* are variable coefficients.^43^ Figure 7(a,d) presents the CV curves recorded at scan rates ranging from 2 to 200 mV/s. To elucidate the charge storage mechanism, the relationship between the logarithm of the peak current (log (*i*)) and the logarithm of the scan rate (log (*v*)) was analyzed, and the corresponding slopes (*b*-value) are shown in Figure 7(b,e). The *b*-value serves as an indicator for identifying the dominant charge storage behavior. A *b*-value approaching 0.5 indicates a diffusion-controlled process, whereas a value close to 1.0 corresponds to capacitive-controlled behavior.^47^ As observed from Figures 7(b,e), NiMoO_4_ NWs/PANI/CS/rGO@CC exhibits a higher *b* value (0.8841) compared to that of NiMoO_4_ NWs/PANI/CS@CC (0.7626), demonstrating that the incorporation of rGO effectively enhances the capacitive contribution of the system.^48^ To quantify the charge storage contributions at different scan rates, a Dunn-type analysis was applied to the CV data. Within the potential window of 0–0.8 V, the measured current was separated into capacitive and diffusion-controlled components. At each potential, the current response was evaluated according to the following relationship

$$i=k_{1}v+k_{2}v^{1/2}$$

$$\frac{i}{v^{1/2}}=k_{1}v^{1/2}+k_{2}$$

where *k*_1_ corresponds to the capacitive-controlled contribution, while *k*_2_ represents the diffusion-controlled contribution.^49^ Linear regression was performed at each potential to extract these parameters, enabling a complete deconvolution of the total current response. Figure 7(c,f) presents the respective contributions of capacitive-controlled and diffusion-controlled processes for NiMoO_4_ NWs/PANI/CS@CC and NiMoO_4_ NWs/PANI/CS/rGO@CC at different scan rates. As the scan rate increases from 2 to 200 mV/s, the capacitive contribution of NiMoO_4_ NWs/PANI/CS@CC gradually rises from 22.7% to 74.6%, while that of NiMoO_4_ NWs/PANI/CS/rGO@CC increases from 35.4% to 84.6%. The enhancement in capacitive contribution with increasing scan rate can be attributed to the dominant surface-controlled charge storage process at high scan rates, where ions have insufficient time to fully undergo diffusion-related adsorption/desorption within the electrode structure.^50^

**Figure 7:**
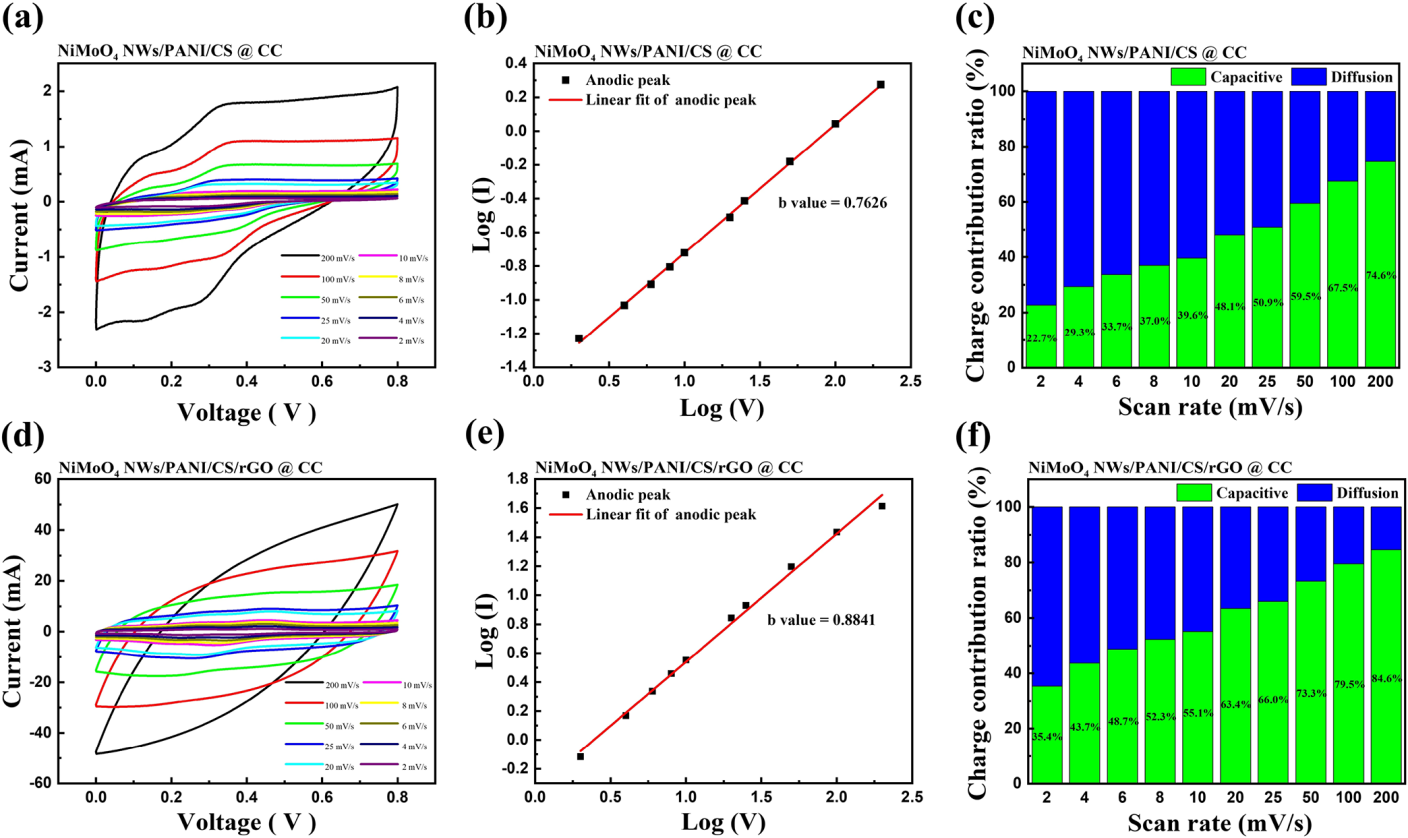
(a, d) CV curves of NiMoO_4_ NWs/PANI/CS@CC and NiMoO_4_ NWs/PANI/CS/rGO@CC at various scan rates within a potential window of 0–0.8 V; (b, e) *b*-value of NiMoO_4_ NWs/PANI/CS@CC and NiMoO_4_ NWs/PANI/CS/rGO@CC; (c, f) capacitive and diffusion-controlled contribution ratios at different scan rates of NiMoO_4_ NWs/PANI/CS@CC and NiMoO_4_ NWs/PANI/CS/rGO@CC.

Figure 8 shows the electrochemical performance of the SSC evaluated by galvanostatic charge–discharge (GCD) measurements at five different constant current, conducted at a voltage of 0.8 V, while Figure S4 presents the corresponding Coulombic efficiency. The areal capacitance (*C*_A_) was calculated using the equation

$$C_{A}=\frac{I\times \Delta t}{A\times \Delta V}$$

where *I* is the applied current, Δ*t* is the discharge time, *A* is the electrode area, and Δ*V* is the potential window.^51^ In this study, GCD tests were performed at constant currents of 4, 2, 1, 0.5, and 0.25 mA. High current densities assess the electrode’s ability to maintain rapid charge–discharge responses and high conductivity, whereas low current densities are ideal for evaluating the material’s energy-storage performance, as it allows adequate time for electrochemical reactions to occur. As shown in Figure 8, the GCD curves exhibit nearly symmetric isosceles triangle shapes at higher currents, indicating typical EDLC behavior.^52^ With decreasing current, the curve shapes change, suggesting that the charge storage mechanism involves not only EDLC but also surface redox reactions. At low currents, the initial sharp potential rise during charging is mainly attributed to the rapid accumulation of charges at the electrode–electrolyte interface. Initially, charges are easily stored in the electric double layer, resulting in a steep potential increase. As charging progresses, additional deposited charges experience Coulombic repulsion from previously diffused charges on the electrode surface, slowing the rate of potential increase.^53^

**Figure 8:**
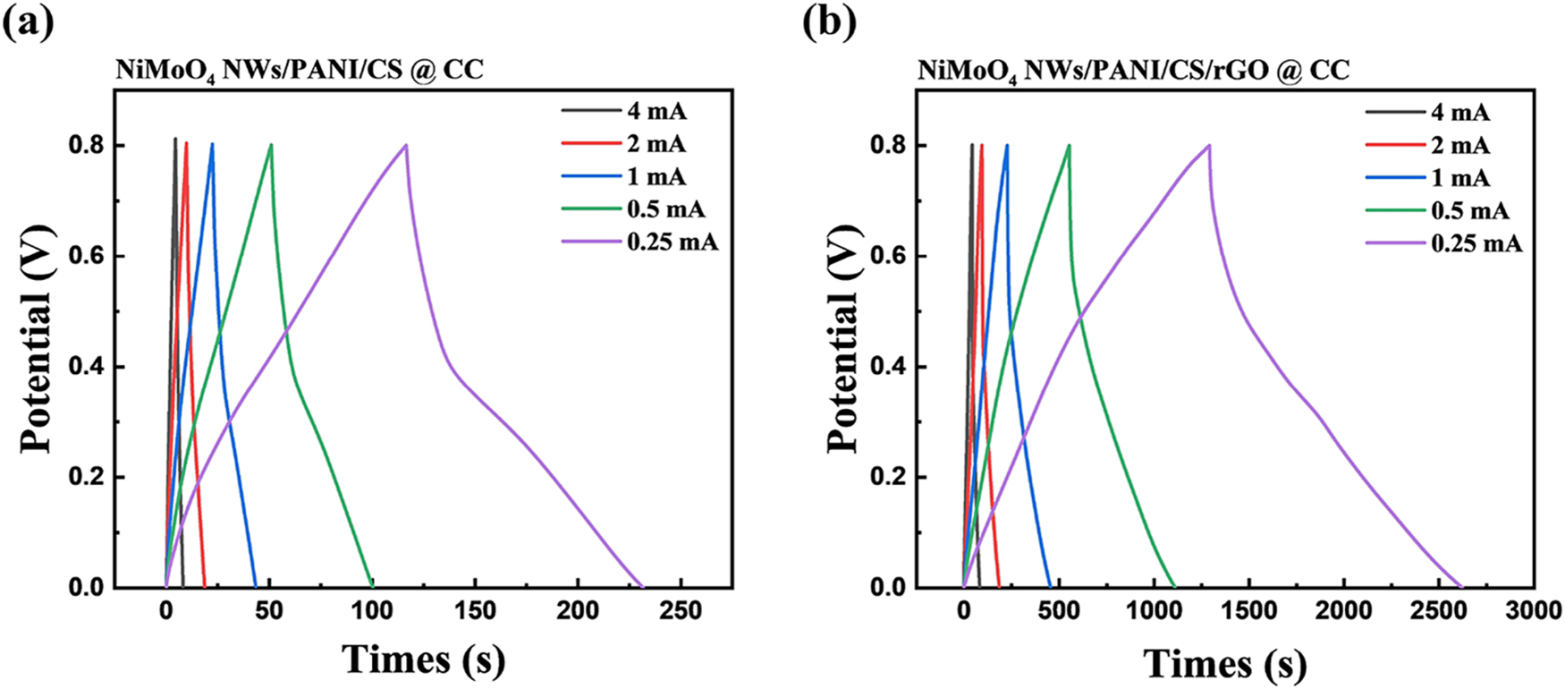
GCD curves of (a) NiMoO_4_ NWs/PANI/CS@CC and (b) NiMoO_4_ NWs/PANI/CS/rGO@CC at different currents of 4, 2, 1, 0.5, and 0.25 mA.

During discharge, the curve can be divided into two regions: the initial near-vertical drop corresponds to the potential drop caused by the internal resistance (IR drop), while the later, more gradual decrease reflects the coexistence of EDLC and pseudocapacitive behavior,^54^ consistent with the CV results. Comparing Figure 8(a,b), the NiMoO_4_ NWs/PANI/CS/rGO@CC electrode exhibits a significantly smaller IR drop, indicating reduced internal mass transport resistance and enhanced ion charge transfer.^55^ Moreover, the longer discharge time results in a higher measured capacitance compared to NiMoO_4_ NWs/PANI/CS@CC, reaching up to 138.49 mF/cm^2^ at 0.25 mA (Table 4). These results further confirm that the incorporation of rGO can effectively enhance the electrochemical performance of the supercapacitor. Such discrepancies between CV- and GCD-derived capacitance values are widely reported in the literature due to their fundamentally different evaluation methods. The capacitance obtained from galvanostatic charge–discharge (GCD) measurements is generally considered more representative of the practical device performance, as it is directly derived from the charge–discharge process under a constant current condition, which closely resembles the actual operation mode of supercapacitor devices. In contrast, cyclic voltammetry (CV) measurements are primarily employed to investigate the electrochemical characteristics and charge storage mechanisms of electrode materials.^56^

**Table 4:** Areal Capacitance of NiMoO_4_ NWs/PANI/CS@CC and NiMoO_4_ NWs/PANI/CS/rGO@CC Calculated from the GCD Curves in Fig. 8

areal capacitance (mF/cm^2^)					
	discharging current (mA)				
sample	4	2	1	0.5	0.25
NiMoO_4_ NWs/PANI/CS@CC	4.50	6.67	8.42	10.08	11.89
NiMoO_4_ NWs/PANI/CS/rGO@CC	64.33	75.83	94.75	116.04	138.49

Figure 9 shows the electrochemical impedance spectroscopy (EIS) results, along with the equivalent circuit used for fitting. The Nyquist plot is the widely used format for presenting EIS data, consisting of the solution resistance (*R*_s_), charge transfer resistance (*R*_ct_), and the Warburg impedance (*W*_o_). The semicircle in the high-frequency region corresponds to the charge transfer resistance at the electrode/electrolyte interface (*R*_ct_), whereas the intercept on the Z′ axis represents the solution resistance (*R*_s_). The linear portion in the low-frequency region is associated with the diffusion of ions and protons at the electrode surface, also referred to as the Warburg impedance (*W*_o_).^57^ The selection of different equivalent circuit models for the two samples is based on the distinct features observed in their Nyquist plots. Specifically, differences in the high-frequency region, including variations in the semicircle shape and diameter, indicate different charge transfer behaviors and interfacial properties. Both models share a common series solution resistance (*R*_s_) connected to a parallel arrangement of charge-transfer resistance (*R*_ct_) and a constant phase element (CPE1). For the NiMoO_4_ NWs/PANI/CS @ CC electrode, the circuit further incorporates a Warburg impedance (*W*_o_) and a pseudocapacitance element (C_1_) in series. In contrast, the NiMoO_4_ NWs/PANI/CS/rGO@CC electrode is modeled with an *R*_ct_ branch that extends into a parallel combination of a second constant phase element (CPE2) and a Warburg impedance (*W*_o_).^58^ Comparing Figure 9(a,b), it can be observed that the NiMoO_4_ NWs/PANI/CS/rGO@CC electrode exhibits a steeper slope in the low-frequency region, indicating superior capacitive performance.^59^ The Warburg resistance parameter (*W*_o_-R) of the NiMoO_4_ NWs/PANI/CS/rGO@CC electrode is 0.612 Ω, which is lower than that of the NiMoO_4_ NWs/PANI/CS@CC electrode (1.734 Ω), indicating enhanced ion transport kinetics. This result is consistent with the steeper slope observed in the low-frequency region, further confirming the improved diffusion behavior of NiMoO_4_ NWs/PANI/CS/rGO@CC. Notably, the NiMoO_4_ NWs/PANI/CS@CC electrode does not show a semicircle in the high-frequency region, which can be attributed to its relatively insulating nature. In such cases, the frequency ranges of effective charge transfer and diffusion overlap, preventing the formation of a complete semicircle in the EIS plot. Moreover, the insulating properties of the coating result in a higher *R*_ct_, leading to surface charging at low frequencies and increased impedance.^60^ After the incorporation of rGO, the enhanced electrical conductivity rendered the charge transfer process more discernible, resulting in a well-defined semicircle. Fitting results (Table 5) show that the *R*_ct_ of NiMoO_4_ NWs/PANI/CS@CC is 1.068 Ω, whereas that of NiMoO_4_ NWs/PANI/CS/rGO@CC is 0.473 Ω, indicating that the incorporation of rGO not only reduces the interfacial resistance but also enhances the charge transfer characteristics.^61,62^

**Figure 9:**
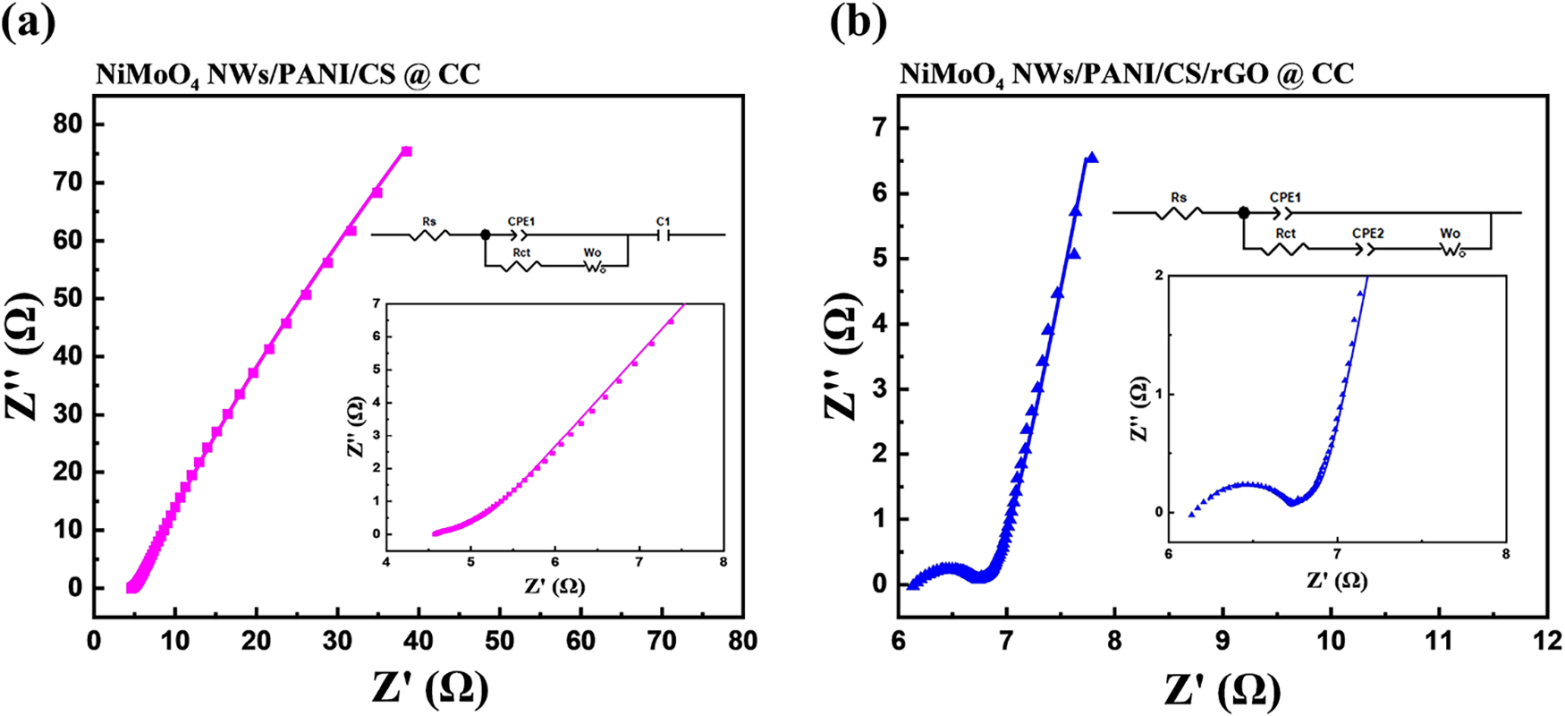
Nyquist plots of (a) NiMoO_4_ NWs/PANI/CS@CC and (b) NiMoO_4_ NWs/PANI/CS/rGO@CC.

**Table 5:** *R*_s_, *R*_ct_, and *W*_o_–*R* values of NiMoO_4_ NWs/PANI/CS@CC and NiMoO_4_ NWs/PANI/CS/rGO@CC Based on Nyquist Plot Fitting Results

EIS analysis			
sample	*R*_s_ (Ω)	*R*_ct_ (Ω)	*W*_o_–*R* (Ω)
NiMoO_4_ NWs/PANI/CS@CC	5.016	1.068	1.734
NiMoO_4_ NWs/PANI/CS/rGO@CC	6.209	0.473	0.612

Figure 10(a) shows the Ragone plot derived from the GCD measurements, which is employed to assess the correlation between the device’s energy density and power density. Tables S4 and 6 summarize the corresponding energy density and power density values, respectively. The energy density and areal power density were calculated according to the following equations

$$E_{A}=\frac{C_{A}\times {\Delta V}^{2}}{7.2}$$

$$P_{A}=\frac{3.6\times E_{A}}{T}$$

**Figure 10:**
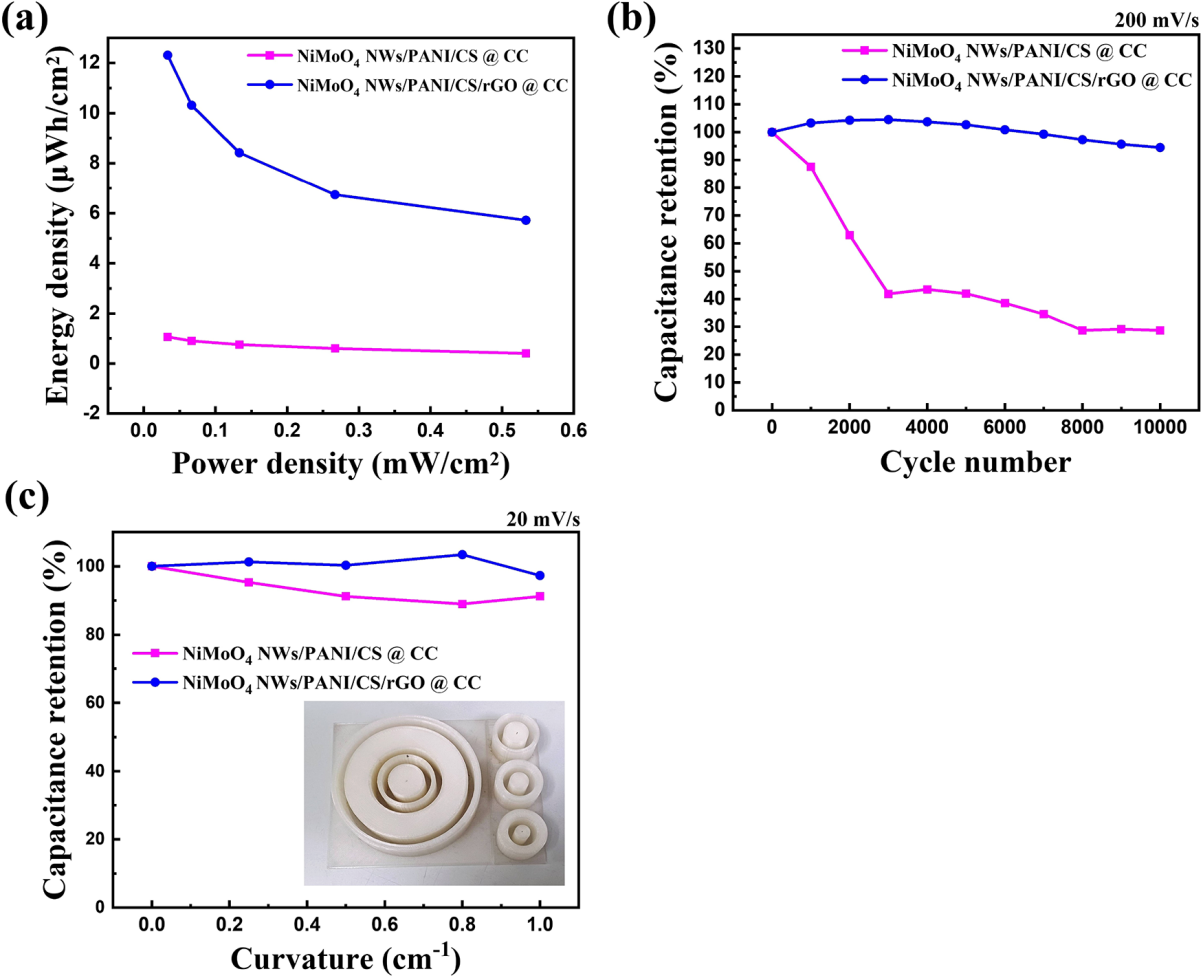
(a) Ragone plots of NiMoO_4_ NWs/PANI/CS@CC and NiMoO_4_ NWs/PANI/CS/rGO@CC. (b) CV cycling test for 10,000 cycles at a potential scan rate of 200 mV/s. (c) Bending test under curvatures of 0–1.0 cm^–1^ with the potential scan rate of 20 mV/s.

**Table 6:** Power Density Derived from GCD Results

power density (mW/cm^2^)					
sample	4 mA	2 mA	1 mA	0.5 mA	0.25 mA
NiMoO_4_ NWs/PANI/CS@CC	0.53	0.27	0.13	0.07	0.03
NiMoO_4_ NWs/PANI/CS/rGO@CC	0.53	0.27	0.13	0.07	0.03

where *E*_A_ is the energy density (μWh/cm^2^), *C*_A_ is the areal capacitance (mF/cm^2^) determined from GCD measurements, Δ*V* is the potential window (*V*), *P*_A_ is the power density (mW/cm^2^), and *T* is the discharge time (s).^63^ The results show that the NiMoO_4_ NWs/PANI/CS/rGO@CC electrode demonstrated a maximum energy density of 12.31 μWh/cm^2^ at a discharge current of 0.25 mA and a highest power density of 0.53 mW/cm^2^ at 4 mA, which are significantly higher than those of the NiMoO_4_ NWs/PANI/CS@CC electrode (1.06 μWh/cm^2^ at 0.25 mA), indicating relatively efficient charge transport and ion diffusion within the electrode structure.^64^ These findings indicate that the energy-storage capability of NiMoO_4_ NWs/PANI/CS/rGO@CC is superior to NiMoO_4_ NWs/PANI/CS@CC. This enhancement can be attributed to the excellent porosity and electrical conductivity of rGO, which effectively improve both the energy density and power density of the supercapacitor.^65^

Figure 10(b) shows the long-term cycling CV test at a scan rate of 200 mV/s, with capacitance retention used to evaluate device performance. The NiMoO_4_ NWs/PANI/CS@CC device exhibited a pronounced decrease in capacitance during the initial cycles, ultimately retaining only ∼30% of its initial capacitance. In contrast, the NiMoO_4_ NWs/PANI/CS/rGO@CC device maintained relatively stable performance throughout the test, with a capacitance retention of 94.5% after 10,000 cycles, demonstrating outstanding long-term durability. Notably, both devices exhibited a slight increase in capacitance retention during cycling, which can be attributed to the continued diffusion of the electrolyte within the porous channels of the composite, gradually activating the electrode material and increasing the number of active sites, thereby enhancing the capacitance.^66^ These results indicate that the incorporation of rGO effectively preserves the structural integrity and electrical conductivity of the composite, preventing aggregation or structural degradation during cycling and ensuring sustained energy-storage performance.^67,68^ Figure 10(c) shows the capacitance retention of the devices at various bending curvatures. CV measurements were conducted at a scan rate of 20 mV/s with curvatures of 0, 0.25, 0.5, 0.8, and 1.0 cm^–1^. The results indicate that the NiMoO_4_ NWs/PANI/CS/rGO@CC device exhibits better bending stability, while both devices show robust overall mechanical performance. Even at a high curvature of 1.0 cm^–1^, the capacitance retention of both devices remains as high as 90–100%, which can be primarily attributed to a mechano-electrochemical synergistic activation effect, where electrolyte ions maintain full contact with the electrode material during repeated bending cycles.^69^ These bending tests not only further confirm the benefits of incorporating rGO but also demonstrate the feasibility of using carbon cloth as a flexible substrate for supercapacitors.

## 4 Conclusion

A flexible symmetric supercapacitor was successfully developed via a cost-effective brush-painting method using a NiMoO_4_ NWs/PANI/CS/rGO composite. The fabricated device demonstrates promising electrochemical performance, mechanical flexibility, and long-term cycling stability, indicating its potential for wearable and portable energy-storage applications. However, flexible symmetric supercapacitors still face inherent limitations, including a relatively restricted operating voltage window and the need for further optimization of ion transport and structural robustness under mechanical deformations. Future work should therefore focus on material and interface engineering and device-level optimization to further enhance energy density and practical applicability.

### 4.1 Key Research Findings

1. Material characterization confirmed the uniform distribution of NiMoO_4_ NWs within the polymer matrix, along with abundant oxygen vacancies and nitrogen-containing functional groups, providing additional active sites for pseudocapacitive reactions.
2. Electrochemical measurements demonstrated that the NiMoO_4_ NWs/PANI/CS/rGO@CC electrode outperformed NiMoO_4_ NWs/PANI/CS@CC in terms of areal capacitance, reduced internal resistance and charge-transfer characteristics.
3. NiMoO_4_ NWs/PANI/CS/rGO@CC achieved a maximum areal capacitance of 138.49 mF/cm^2^ at a current of 0.25 mA, corresponding to a peak energy density of 12.31 μWh/cm^2^.
4. The *b*-value analysis showed that NiMoO_4_ NWs/PANI/CS/rGO@CC (*b* = 0.8841) exhibits a more pronounced capacitive behavior compared to NiMoO_4_ NWs/PANI/CS@CC (*b* = 0.7626), indicating that the incorporation of rGO enhances capacitive-controlled charge storage. Further quantitative separation revealed that the capacitive contribution increases with scan rate for both electrodes, reaching up to 84.6% for NiMoO_4_ NWs/PANI/CS/rGO@CC at 200 mV/s.
5. NiMoO_4_ NWs/PANI/CS/rGO@CC exhibited excellent long-term cycling performance, maintaining 94.5% of its original capacitance even after 10,000 cycles, as well as outstanding mechanical flexibility under various bending curvatures.

## Supplementary Material



## Notes

EDS spectra of NiMoO_4_ NWs/PANI/CS/rGO@CC, (Figure S1); XPS N 1s spectra of NiMoO_4_ NWs/PANI/CS/rGO@CC, (Figure S2); water contact angle of NiMoO_4_ NWs/PANI/CS@CC, (Figure S3); coulombic efficiency of NiMoO_4_ NWs/PANI/CS@CC and NiMoO_4_ NWs/PANI/CS/rGO@CC, (Figure S4); comparison of representative fabrication methods for supercapacitor electrodes, (Table S1); atomic ratios of NiMoO_4_ NWs/PANI/CS/rGO@CC from (Figure S1 and Table S2); surface atomic ratios of NiMoO_4_ NWs/PANI/CS/rGO@CC, (Table S3); energy density derived from GCD results, (Table S4); comparison of electrochemical performance of recently reported flexible supercapacitors based on NiMoO_4_, rGO/PANI, and related systems, (Table S5) (PDF)

## References

[1] Yu Z., Tang D. (2022). Artificial neural network-assisted
wearable flexible sweat patch
for drug management in Parkinson’s patients based on vacancy-engineered
processing of g-C3N4. Anal. Chem..

[2] Wu D., Tang J., Yu Z., Gao Y., Zeng Y., Tang D., Liu X. (2024). Pt/Zn-TCPP
nanozyme-based flexible
immunoassay for dual-mode pressure–temperature monitoring of
low-abundance proteins. Anal. Chem..

[3] Vandeginste V. (2022). A review of
fabrication technologies for carbon electrode-based micro-supercapacitors. Appl. Sci..

[4] Sasikumar K., Ju H. (2022). Recent progress in the core-shell nanostructures of the NiMoO4-based
composite materials for supercapacitor applications: a comprehensive
review. Chemosensors.

[5] An C., Zhang Y., Guo H., Wang Y. (2019). Metal oxide-based supercapacitors:
progress and prospectives. Nanoscale Adv..

[6] Low W. H., Khiew P. S., Lim S. S., Siong C. W., Ezeigwe E. R. (2019). Recent
development of mixed transition metal oxide and graphene/mixed transition
metal oxide based hybrid nanostructures for advanced supercapacitors. J. Alloys Compd..

[7] Zhang Y., Chang C.-r., Jia X.-d., Huo Q.-y., Gao H.-l., Yan J., Zhang A.-q., Ru Y., Mei H.-x., Gao K.-z., Wang L. z. (2020). Morphology-dependent
NiMoO4/carbon composites for high
performance supercapacitors. Inorg. Chem. Commun..

[8] Murugan E., Govindaraju S., Santhoshkumar S. (2021). Hydrothermal synthesis, characterization
and electrochemical behavior of NiMoO4 nanoflower and NiMoO4/rGO nanocomposite
for high-performance supercapacitors. Electrochim.
Acta.

[9] Moosavifard S. E., Shamsi J., Ayazpour M. (2015). 2D high-ordered
nanoporous NiMoO4
for high-performance supercapacitors. Ceram.
Int..

[10] Hsin J.-C., Cheng Y.-C., Wang M.-J., Hsu C.-C., Cheng I.-C., Chen J.-Z. (2020). Ar dielectric barrier
discharge jet (DBDjet) plasma
treatment of reduced graphene oxide (rGO)–polyaniline (PANI)–chitosan
(CS) nanocomposite on carbon cloth for supercapacitor application. Energy, Ecol. Environ..

[11] Pandiselvi K., Thambidurai S. (2014). Chitosan-ZnO/polyaniline
ternary nanocomposite for
high-performance supercapacitor. Ionics.

[12] Kovalenko I., Bucknall D. G., Yushin G. (2010). Detonation nanodiamond and onion-like-carbon-embedded
polyaniline for supercapacitors. Adv. Funct.
Mater..

[13] Yu P., Li Y., Zhao X., Wu L., Zhang Q. (2014). Graphene-wrapped polyaniline
nanowire arrays on nitrogen-doped carbon fabric as novel flexible
hybrid electrode materials for high-performance supercapacitor. Langmuir.

[14] Zhou Q., Lin Y., Zhang K., Li M., Tang D. (2018). Reduced graphene oxide/BiFeO3
nanohybrids-based signal-on photoelectrochemical sensing system for
prostate-specific antigen detection coupling with magnetic microfluidic
device. Biosens. Bioelectron..

[15] Vellakkat M., Hundekal D. (2017). Electrical conductivity
and supercapacitor properties
of polyaniline/chitosan/nickel oxide honeycomb nanocomposite. J. Appl. Polym. Sci..

[16] Lim S.-H., Sim H.-M., Kim G., Kim H.-K. (2021). Brush-paintable
black electrodes for poly (vinylidene fluoride)-based flexible piezoelectric
devices. ACS Omega.

[17] Miya L. A., Ghosh S. K., Kumari P., Morema C. N. M., Mallick K. (2025). Eco-friendly
and sustainable supercapacitor design: Cobalt sulfide nanoparticles
embedded on carbon cloth as an electrode material for asymmetric devices. Chem. Pap..

[18] Govindasamy M., Shanthi S., Elaiyappillai E., Wang S.-F., Johnson P. M., Ikeda H., Hayakawa Y., Ponnusamy S., Muthamizhchelvan C. (2019). Fabrication of hierarchical NiCo2S4@
CoS2 nanostructures
on highly conductive flexible carbon cloth substrate as a hybrid electrode
material for supercapacitors with enhanced electrochemical performance. Electrochim. Acta.

[19] Bhosale S. V., Bhosale S. V. (2025). Advancements in
supercapacitors: breaking barriers
and enabling amazing applications. Chem. Sci..

[20] Momeni M.
M., Mohammadinejad F., Ghasemipur F., Lee B.-K. (2025). Asymmetric photo-assisted
supercapacitor and symmetric flexible supercapacitors based on CeO2-MnO2
supported on carbon cloth. J. Alloys Compd..

[21] Chiu L.-D., Yu S.-E., Chueh C.-C., Ni I.-C., Wu C.-I., Cheng I.-C., Chen J.-Z. (2025). NiMoO4
nanowires supported on stainless-steel,
carbon, and nickel fiber papers as catalysts for the oxygen evolution
reaction in anion exchange membrane water electrolysis. ACS Appl. Nano Mater..

[22] Kumar A. M., Jose J., Hussein M. A. (2022). Novel polyaniline/chitosan/reduced
graphene oxide ternary nanocomposites: Feasible reinforcement in epoxy
coatings on mild steel for corrosion protection. Prog. Org. Coat..

[23] Hosseini M. G., Shahryari E. (2017). A novel high-performance
supercapacitor based on chitosan/graphene
oxide-MWCNT/polyaniline. J. Colloid Interface
Sci..

[24] He C., Yang G., Ni L., Yang H., Peng Y., Liu X., Li P., Song C., He S., Zhang Q. (2024). N/O Co-doped
porous carbon with controllable porosity synthesized via an all-in-one
step method for a high-rate-performance supercapacitor. Langmuir.

[25] Gao W., Zhang F., Zhang S., Li J.-y., Lian H.-z. (2023). Hydrophilic
porous magnetic graphene oxide/chitosan composites for the selective
separation and enrichment of N-glyco-and phosphopeptides. ACS Appl. Nano Mater..

[26] Minitha C. R., Anithaa V. S., Subramaniam V., Kumar R. T. R. (2018). Impact of oxygen
functional groups on reduced graphene oxide-based sensors for ammonia
and toluene detection at room temperature. ACS
Omega.

[27] Jiang C., Zhang X., Lu S., Zhang W., Wang J., Yan X., Zhao X., Liu B. (2023). Electrochemical
Performance of Oxygen
Vacancies Enhanced Transition Metal Oxides in Supercapacitor. J. Chin. Ceram. Soc..

[28] Hsu A. R., Chien H.-H., Liao C.-Y., Lee C.-C., Tsai J.-H., Hsu C.-C., Cheng I.-C., Chen J.-Z. (2018). Scan-mode
atmospheric-pressure
plasma jet processed reduced graphene oxides for quasi-solid-state
gel-electrolyte supercapacitors. Coatings.

[29] Zhang C., Liu T., Lu B. (2025). Electrocatalytic
CO2 Reduction over Pyridinic Nitrogen-Doped
Carbon as a Metal-Free Catalyst. Langmuir.

[30] Yang W., Peng D., Kimura H., Zhang X., Sun X., Pashameah R. A., Alzahrani E., Wang B., Guo Z., Du W., Hou C. (2022). Honeycomb-like nitrogen-doped porous carbon decorated
with Co3O4 nanoparticles for superior electrochemical performance
pseudo-capacitive lithium storage and supercapacitors. Adv. Compos. Hybrid Mater..

[31] Chen C., Wang S., Luo X., Gao W., Huang G., Zeng Y., Zhu Z. (2019). Reduced ZnCo2O4@ NiMoO4·
H2O
heterostructure electrodes with modulating oxygen vacancies for enhanced
aqueous asymmetric supercapacitors. J. Power
Sources.

[32] Yang Y., Ma Y., Sun C., Bu C., Yan Y., Li X. (2025). Porous NiMoO4@
NiMn-LDH core–shell nanocomposites anchored on nickel foam:
application in asymmetric supercapacitors with a high specific capacitance. ACS Appl. Energy Mater..

[33] Zhang H., Lu C., Hou H., Ma Y., Yuan S. (2019). Tuning the electrochemical
performance of NiCo2O4@ NiMoO4 core-shell heterostructure by controlling
the thickness of the NiMoO4 shell. Chem. Eng.
J..

[34] Xu H., Zhang L., Wang A., Hou J., Guo X. (2021). Facile preparation
of oxygen-vacancy-engineered MoOx nanostructures for photoreversible
switching systems. Nanomaterials.

[35] Li M., Zhao X., Xu Y., Yan Z. C., Wei H., Wang Y., Yang H. Y. (2025). A mini-review
about overcoming challenges
in hydrophilicity: Towards efficient capacitive deionization electrodes. Sep. Purif. Technol..

[36] Adotey E., Kurbanova A., Ospanova A., Ardakkyzy A., Toktarbay Z., Kydyrbay N., Zhazitov M., Nuraje N., Toktarbaiuly O. (2025). Development of superhydrophobic reduced graphene oxide
(rGO) for potential applications in advanced materials. Nanomater..

[37] Zhou Y., Ning X. (2024). Improving Wettability at Positive Electrodes to Enhance the Cycling
Stability of Bi-Based Liquid Metal Batteries. Small.

[38] Yu T. T., Liu H., Huang M., Zhang J., Su D., Tang Z., Xie J., Liu Y., Yuan A., Kong Q. (2017). Zn 2 GeO 4 nanorods
grown on carbon cloth as high performance flexible lithium-ion battery
anodes. RSC Adv..

[39] Fouzia K., Seema H., AbalKhail A. A., Khan S., Shahab A., Malik M. O., Almutlaq F. (2026). A Novel Polyaniline
Gadolinium Oxide
Coated Reduced Graphene Oxide Nanocomposite: A Sustainable, Cost-Effective
and High-Performance Counter Electrode for Dye-Sensitized Solar Cells. Catalysts.

[40] Kosowska K., Domalik-Pyzik P., Nocuń M., Chłopek J. (2018). Chitosan and
graphene oxide/reduced graphene oxide hybrid nanocomposites–Evaluation
of physicochemical properties. Mater. Chem.
Phys..

[41] Zhao Y., Liu L., Cui T., Tong G., Wu W. (2017). Enhanced photocatalytic
properties of ZnO/reduced graphene oxide sheets (rGO) composites with
controllable morphology and composition. Appl.
Surf. Sci..

[42] Wu Z., Guo C., Lu Z., Yuan C., Xu Y., Dai L. (2023). A facile brushing
method for constructing all-in-one high performance flexible supercapacitor
with ordinary carbon materials. J. Energy Storage.

[43] Mohamad A. A. (2025). Cyclic
voltammetry of hybrid supercapacitors: A characterization review. Inorg. Chem. Commun..

[44] Czagany M., Hompoth S., Keshri A. K., Pandit N., Galambos I., Gacsi Z., Baumli P. (2024). Supercapacitors: An
efficient way
for energy storage application. Mater..

[45] Aderyani S., Flouda P., Shah S., Green M., Lutkenhaus J., Ardebili H. (2021). Simulation of cyclic
voltammetry in structural supercapacitors
with pseudocapacitance behavior. Electrochim.
Acta.

[46] Chang J.-H., Chen S.-Y., Kuo Y.-L., Yang C.-R., Chen J.-Z. (2021). Carbon
dioxide tornado-type atmospheric-pressure-plasma-jet-processed rGO-SnO2
nanocomposites for symmetric supercapacitors. Materials.

[47] Utturkar V., Kaka F. (2026). An electrochemical modeling framework for estimating charge storage
behavior: Evaluating effects of nanomaterials with experimental validation. J. Energy Storage.

[48] Sami A., Khan M. M., Haidar Z., Bahajjaj A. A. A., Riaz S., Naseem S., Osman S. M., Khan M. S. (2025). Enhanced supercapacitor
performance of rGO-Supported BaVO3 nanostructures as advanced electrode
materials. Ceram. Int..

[49] Babu C. R., Anila E., Avani A., Jose R., S X. T. (2026). An investigation
on the electrochemical performance of Mn 3 O 4-based aqueous symmetric
supercapacitor devices. RSC Adv..

[50] Hassan H., Iqbal M., Alrobei H., Riasat F., Afzal A., Saeedi A., Albargi H., Rehmat A. (2024). Synergistic CuCoS–PANI
materials for binder-free electrodes in asymmetric supercapacitors
and oxygen evolution. Nanoscale Adv..

[51] Prasad A. K., Park J.-Y., Kang S.-H., Ahn K.-S. (2022). Electrochemically
co-deposited WO3-V2O5 composites for electrochromic energy storage
applications. Electrochim. Acta.

[52] Ngamjumrus N., Silakaew K., Thompho S., Sriwong C., Ruttanapun C. (2023). Two Steps
for improving reduced graphene oxide/activated durian shell carbon
composite by hydrothermal and 3-D ball milling process for symmetry
supercapacitor device. Energy.

[53] Elmanfaloty R. A., Shokry E., Abou-bakr E., Ebrahim S., Elshaer A. (2024). Electrochemical
measurements, structural and morphological studies of electrodeposited
polypyrrole supercapacitor electrode. Alexandria
Eng. J..

[54] Gund G. S., Dubal D. P., Patil B. H., Shinde S. S., Lokhande C. D. (2013). Enhanced
activity of chemically synthesized hybrid graphene oxide/Mn3O4 composite
for high performance supercapacitors. Electrochim.
Acta.

[55] Guan X., Pan L., Fan Z. (2021). Flexible, transparent and highly conductive polymer
film electrodes for all-solid-state transparent supercapacitor applications. Membranes.

[56] Yu L., Chen G. Z. (2020). Supercapatteries as high-performance electrochemical
energy storage devices. Electrochem. Energy
Rev..

[57] Kaipannan S., Marappan S. (2019). Fabrication of 9.6 V high-performance
asymmetric supercapacitors
stack based on nickel hexacyanoferrate-derived Ni (OH) 2 nanosheets
and bio-derived activated carbon. Sci. Rep.

[58] Hakamy A. (2025). Investigation
of double-layer capacitance, Warburg finite-length impedance and AC
conductivity of PVA/MWCNT nanocomposite films for supercapacitor applications. J. Power Sources.

[59] Saha D., Li Y., Bi Z., Chen J., Keum J. K., Hensley D. K., Grappe H. A., Meyer III H. M., Dai S., Paranthaman M. P., Naskar A. K. (2014). Studies on supercapacitor electrode material from activated
lignin-derived mesoporous carbon. Langmuir.

[60] Randviir E. P., Banks C. E. (2022). A review of electrochemical
impedance spectroscopy
for bioanalytical sensors. Anal. Methods.

[61] Wang H., Xu M., Wu D., Tie D., Li M., Tang D. (2026). Built-In Electric
Field-Assisted Enzyme Catalytic Amplification with ZnO/CIS Heterojunctions
for Photoelectrochemical Immunoassay. Anal.
Chem..

[62] Wang H., Tang J., Wan X., Wang X., Zeng Y., Liu X., Tang D. (2024). Mechanism exploration
of the photoelectrochemical immunoassay
for the integration of radical generation with self-quenching. Anal. Chem..

[63] Zhang Y., Liu Y., Bai Y., Liu Y., Xie E. (2020). Boosting the electrochemical
properties of carbon materials as bipolar electrodes by introducing
oxygen functional groups. RSC Adv..

[64] Wang X., Mehandzhiyski A. Y., Arstad B., Van Aken K. L., Mathis T. S., Gallegos A., Tian Z., Ren D., Sheridan E., Grimes B. A. (2017). Selective charging behavior in an ionic mixture
electrolyte-supercapacitor system for higher energy and power. J. Am. Chem. Soc..

[65] Jha P. K., Singh S. K., Kumar V., Rana S., Kurungot S., Ballav N. (2017). High-level supercapacitive performance of chemically
reduced graphene oxide. Chem.

[66] Gul H., Shah A.-u.-H. A., Bilal S. (2019). Achieving ultrahigh cycling stability
and extended potential window for supercapacitors through asymmetric
combination of conductive polymer nanocomposite and activated carbon. Polymer.

[67] Zhang Q., Zhou Y., Tong Y., Chi Y., Liu R., Dai C., Li Z., Cui Z., Liang Y., Tan Y. (2023). Reduced graphene
oxide coating LiFePO4 composite cathodes for advanced lithium-ion
battery applications. Int. J. Mol. Sci..

[68] Zhu Y., Cheng S., Zhou W., Jia J., Yang L., Yao M., Wang M., Zhou J., Wu P., Liu M. (2017). Construction
and performance characterization of α-Fe2O3/rGO composite for
long-cycling-life supercapacitor anode. ACS
Sustainable Chem. Eng..

[69] Navaneeth P., Kumar A., Nair B. G., TG S. B., Suneesh P. V. (2022). Studies on fabrication
of high-performance flexible printed supercapacitor
using cobalt hydroxide nanowires. Electrochim.
Acta.

