# Supplementary material for: Performance Improvement by Adding Reduced Graphene Oxides in Brush-Painted NiMoO4 Nanowires–Polyaniline–Chitosan Composite Flexible Supercapacitor
Source: Langmuir. 2026 Jun 22;42(26):19015–28. doi: 10.1021/acs.langmuir.6c01763 (PMC13352633; doi:10.1021/acs.langmuir.6c01763)
Supplement: Supplementary file 1 [file la6c01763_si_001.pdf]

## Supplementary Information

# Performance improvement by adding reduced graphene oxides in brush-painted $NiMoO_4$ nanowires-polyaniline-chitosan composite flexible supercapacitor

*Pei-Yi Yan[1], Lu-Hong Chen[2], Li-Da Chiu[1], Yi-Der Huang[2], I-Chun Cheng[4], Jian-Zhang Chen[1,2,3,5*]*

*[1] Graduate School of Advanced Technology, National Taiwan University, Taipei City 106319, Taiwan*

*[2] Institute of Applied Mechanics, National Taiwan University, Taipei City 106319, Taiwan*

*[3] Advanced Research Center for Green Materials Science and Technology, National Taiwan University, Taipei City 106319, Taiwan*

*[4]Graduate Institute of Photonics and Optoelectronics and Department of Electrical Engineering, National Taiwan University, Taipei City 106319, Taiwan*

*[5]Research Center for Applied Sciences, Academia Sinica, Taipei City 115201, Taiwan*

*[*] Correspondence: jchen@ntu.edu.tw; Tel.: +886-2-3366-5694*

**List:**

1. **Fig. S1.** EDS spectra of $NiMoO_4$ NWs/PANI/CS/rGO @ CC.
2. **Fig. S2.** XPS N1s spectra of $NiMoO_4$ NWs/PANI/CS/rGO @ CC.
3. **Fig. S3.** Water contact angle of $NiMoO_4$ NWs/PANI/CS @ CC.
4. **Fig. S4.** Coulombic efficiency of $NiMoO_4$ NWs/PANI/CS @ CC and $NiMoO_4$ NWs/PANI/CS/rGO @ CC.
5. **Table S1.** Comparison of representative fabrication methods for supercapacitor electrodes.
6. **Table S2.** Atomic ratios of $NiMoO_4$ NWs/PANI/CS/rGO @ CC from Fig. S1.
7. **Table S3.** Surface atomic ratios of $NiMoO_4$ NWs/PANI/CS/rGO @ CC.
8. **Table S4.** Energy density derived from GCD results.
9. **Table S5.** Comparison of electrochemical performance of recently reported flexible supercapacitors based on $NiMoO_4$, rGO/PANI, and related systems.

**Table S1.** Comparison of representative fabrication methods for supercapacitor electrodes.

| Fabrication Method | Cost | Substrate Compatibility | Throughput | Key Features | Reference |
|---|---|---|---|---|---|
| Brush-painted | Low | Flexible substrates (e.g., fabric/carbon cloth) | Moderate | Simple, ambient, no special equipment | 1 |
| CVD | High | Limited (no polymers) | Low | High cost, rigid substrates | 2 |
| Screen printing | Low–Medium | Paper, cloth, PET | High | Precise patterns | 2 |
| Spray coating | Low–Medium | Flexible/ wearable substrates | High | Uniform thin films, scalable | 3 |
| 3D printing | Medium | Many | Medium | Versatile 3D structures | 2, 4 |
| Magnetron sputtering | High | Mainly rigid substrates; limited flexibility | Low–Medium | Uniform thin films; vacuum system | 5 |

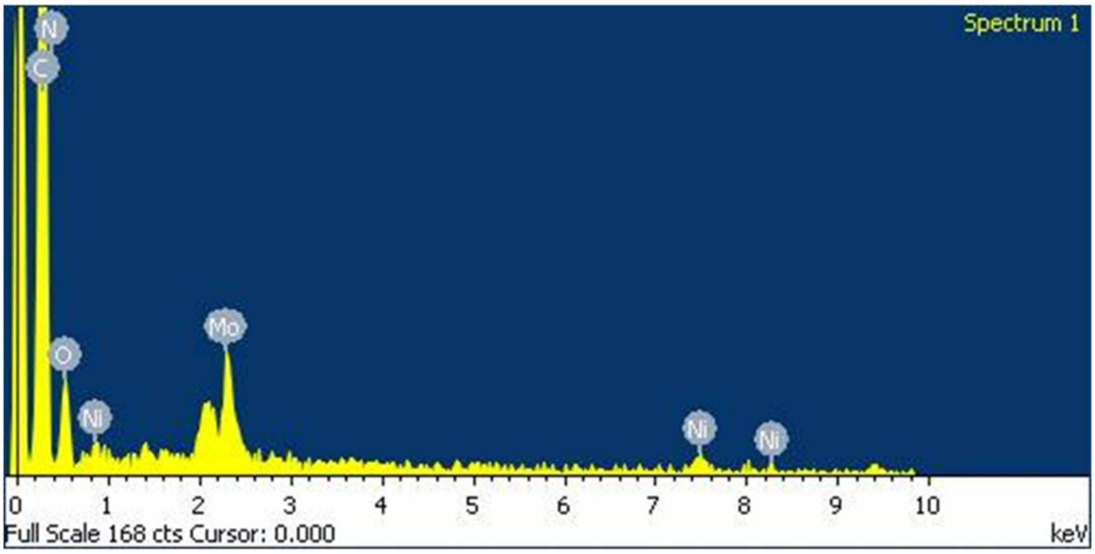

**Fig. S1.** EDS spectra of $NiMoO_4$ NWs/PANI/CS/rGO @ CC.

**Table S2.** Atomic ratios of $NiMoO_4$ NWs/PANI/CS/rGO @ CC from Fig. S1.

| **Atomic ratio (%)** | | | | | |
|---|---|---|---|---|---|
| Ni | Mo | C | O | N | Totals |
| 1.09 | 1.25 | 85.10 | 11.62 | 0.95 | 100 |

**Table S3.** Surface atomic ratios of $NiMoO_4$ NWs/PANI/CS/rGO @ CC.

| **Atomic ratio (%)** | | | | | |
|---|---|---|---|---|---|
| Ni | Mo | C | O | N | Totals |
| 1.42 | 2.28 | 56.46 | 30.83 | 9.01 | 100 |

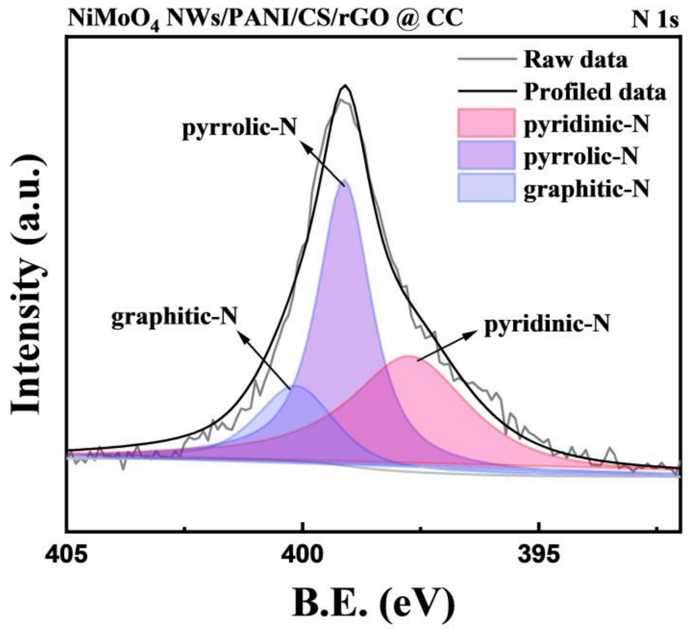


**Fig. S2.** XPS N1s spectra of $NiMoO_4$ NWs/PANI/CS/rGO @ CC.

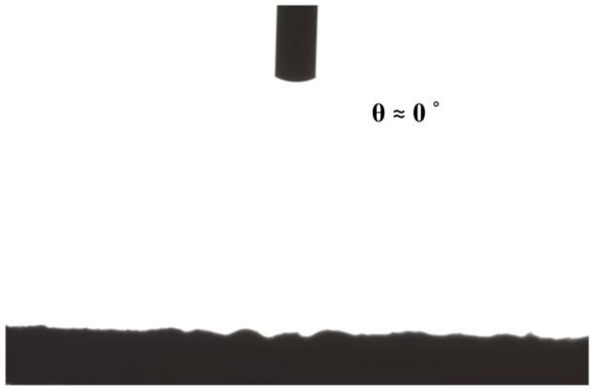


**Fig. S3.** Water contact angle of $NiMoO_4$ NWs/PANI/CS @ CC.

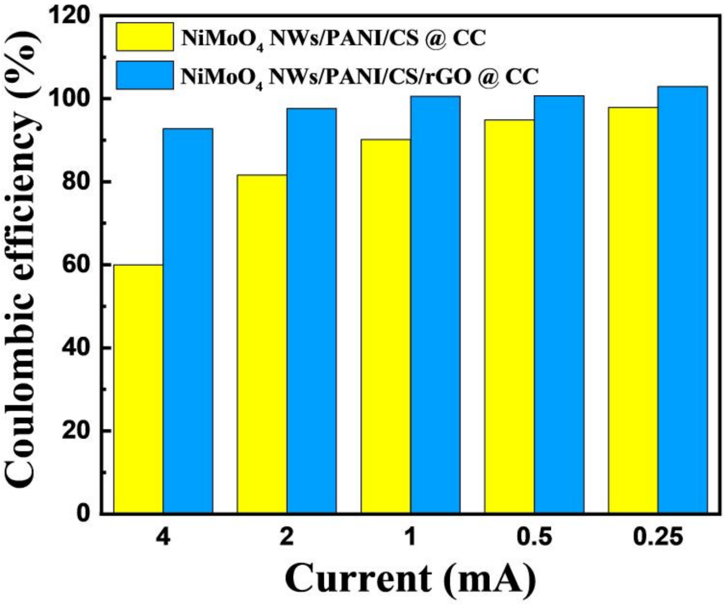


**Fig. S4.** Coulombic efficiency of $NiMoO_4$ NWs/PANI/CS @ CC and $NiMoO_4$ NWs/PANI/CS/rGO @ CC.

**Table S4.** Energy density derived from GCD results.

| **Energy density (μWh/cm²)** | | | | | |
|---|---|---|---|---|---|
| Sample | 4 mA | 2 mA | 1 mA | 0.5 mA | 0.25 mA |
| $NiMoO_4$ NWs/PANI/CS @ CC | 0.40 | 0.59 | 0.75 | 0.90 | 1.06 |
| $NiMoO_4$ NWs/PANI/CS/rGO @ CC | 5.72 | 6.74 | 8.42 | 10.31 | 12.31 |

**Table S5.** Comparison of electrochemical performance of recently reported flexible supercapacitors based on $NiMoO_4$, rGO/PANI, and related systems.

| Electrode materials | Electrolyte | CV capacitance (mF/cm²) | GCD capacitance (mF/cm²) | Energy density | Cycling stability (%) | Bending stability (%) | Reference |
|---|---|---|---|---|---|---|---|
| $NiMoO_4$ NWs/PANI/CS/rGO @ CC | 1 M $H_2SO_4$ | 125.85 | 138.49 | 12.31 µWh/cm² | 94.5 (10,000 cycles) | 97.3 | This work |
| $NiMoO_4$/NiMn-LDH @ NF // AC | 3 M KOH | - | 9438.4 | 1200 µWh/cm² | 95 (7000 cycles) | - | [6] |
| rZCO/$NiMoO_4\cdot H_2O$ NWs @ NF // CNT | 1 M KOH | - | 3530 | 2550 µWh/cm³ | 80 (5000 cycles) | - | [7] |
| $NiCo_2O_4$/$NiMoO_4$ @ CC // AC | 6 M KOH | - | 2522 | 53.3 Wh/kg | 89.8 (5000 cycles) | - | [8] |
| $NiCo_2O_4$/$NiMoO_4$/PANI @ CC (symmetric) | 1 M $H_3PO_4$ | - | 2380 | 90 Wh/kg | 92.36 (5000 cycles) | 89.4 | [9] |
| $NiMoO_4$/PANI @ CC // AC | 1 M KOH | - | 1340 | 99.26 Wh/kg | 94.9 (20,000 cycles) | 90.2 | [10] |
| $NiMoO_4$/NiO @ NF // AC | 3 M KOH | - | 10300 | 38 Wh/kg | 95.5 (5000 cycles) | - | [11] |
| rGO/PANI @ graphite sheet (symmetric) | 1 M $H_2SO_4$ | 28.37 | 15.81 | - | 86.9 (1000 cycles) | - | [12] |
| PANI @ graphene paper (symmetric) | 0.5 M $H_2SO_4$ | 123 | 176 | 17.1 µWh/cm² | - | 74.8 | [13] |

| | | | | | | | |
|---|---|---|---|---|---|---|---|
| rGO/PANI @ CC (three-electrode system) | 1 M $H_2SO_4$; 1 M $Li_2SO_4$ | 55.30 (1 M $H_2SO_4$) | 48.33 (1 M $H_2SO_4$) | - | 74.61 (10000 cycles; 1 M $Li_2SO_4$) | - | [14] |
| rGO/PANI/CS @ black ink-coated paper | 1 M $H_2SO_4$ | 110.63 | 179.08 | - | - | Stable (1000 cycles, no % given) | [15] |
| PVA/CNT/PANI @ CC (symmetric) | 1 M $H_2SO_4$ | 196.5 | - | - | 71.4 (5000 cycles) | - | [16] |
| rGO/PPy/CS @ CC (symmetric) | 1 M $H_2SO_4$ | 45.32 | 72.79 | 6.5 | 88 (10000 cycles) | Stable (no % reported) | [17] |
| rGO/LiMnOx @ CC // rGO | 1M $Li_2SO_4$ | 138.91 | 117.35 | 9.4 µWh/cm² | 96 (4000 cycles) | 89 | [18] |
| ZnCo@SSP//rGO@CC | 1M KOH | 51.73 | 40.25 | 12.33 µWh/cm² | 74 (5000 cycles) | 160 | [19] |

## References


(1) Wu, Z.; Guo, C.; Lu, Z.; Yuan, C.; Xu, Y.; Dai, L. A facile brushing method for constructing all-in-one high performance flexible supercapacitor with ordinary carbon materials. *J. Energy Storage* **2023**, *67*, 107531.

(2) Vandeginste, V. A review of fabrication technologies for carbon electrode-based micro-supercapacitors. *Appl. Sci.* **2022**, *12* (2), 862.

(3) Say, M. G.; Brooke, R.; Edberg, J.; Grimoldi, A.; Belaineh, D.; Engquist, I.; Berggren, M. Spray-coated paper supercapacitors. *npj Flexible Electron.* **2020**, *4* (1), 14.

(4) Jha, S.; Velhal, M.; Stewart, W.; Amin, V.; Wang, E.; Liang, H. Additively manufactured electrodes for supercapacitors: a review. *Appl. Mater. Today* **2022**, *26*, 101220.

(5) Cheng, J.; Mi, B.; Wang, Q.; Wang, H.; Zhou, T.; Li, Y.; Hou, H.; Zhu, Y. Research on magnetron sputtering thin films as electrode materials for supercapacitors. *Chem. Eng. J.* **2025**, *509*, 161242.

(6) Yang, Y.; Ma, Y.; Sun, C.; Bu, C.; Yan, Y.; Li, X. Porous NiMoO4@ NiMn-LDH core–shell nanocomposites anchored on nickel foam: application in asymmetric supercapacitors with a high specific capacitance. *ACS Appl. Energy Mater.* **2025**, *8* (8), 5110–5122.

(7) Chen, C.; Wang, S.; Luo, X.; Gao, W.; Huang, G.; Zeng, Y.; Zhu, Z. Reduced ZnCo2O4@ NiMoO4· H2O heterostructure electrodes with modulating oxygen vacancies for enhanced aqueous asymmetric supercapacitors. *J. Power Sources* **2019**, *409*, 112–122.

(8) Zhang, H.; Lu, C.; Hou, H.; Ma, Y.; Yuan, S. Tuning the electrochemical performance of NiCo2O4@ NiMoO4 core-shell heterostructure by controlling the thickness of the NiMoO4 shell. *Chem. Eng. J.* **2019**, *370*, 400–408.

(9) Shen, J.; Wang, Q.; Zhang, K.; Wang, S.; Li, L.; Dong, S.; Zhao, S.; Chen, J.; Sun, R.; Wang, Y. Flexible carbon cloth based solid-state supercapacitor from hierarchical holothurian-morphological NiCo2O4@ NiMoO4/PANI. *Electrochim. Acta* **2019**, *320*, 134578.

(10) Chen, Y.; Liu, B.; Liu, Q.; Wang, J.; Liu, J.; Zhang, H.; Hu, S.; Jing, X. Flexible all-solid-state asymmetric supercapacitor assembled using coaxial NiMoO4 nanowire arrays with chemically integrated conductive coating. *Electrochim. Acta* **2015**, *178*, 429–438.

(11) Xu, R.; Lin, J.; Wu, J.; Huang, M.; Fan, L.; Xu, Z.; Song, Z. A high-performance pseudocapacitive electrode material for supercapacitors based on the unique NiMoO4/NiO nanoflowers. *Appl. Surf. Sci.* **2019**, *463*, 721–731.

(12) Hao, Y.-C.; Nurzal, N.; Chien, H.-H.; Liao, C.-Y.; Kuok, F.-H.; Yang, C.-C.; Chen, J.-Z.; Yu, I.-S. Application of atmospheric-pressure-plasma-jet modified flexible graphite

sheets in reduced-graphene-oxide/polyaniline supercapacitors. *Polym.* **2020**, *12* (6), 1228.
(13) Li, K.; Liu, X.; Chen, S.; Pan, W.; Zhang, J. A flexible solid-state supercapacitor based on graphene/polyaniline paper electrodes. *J. Energy Chem.* **2019**, *32*, 166–173.
(14) Cho, W.-H.; Cheng, I.-C.; Chen, J.-Z. Performance comparison of reduced graphene oxide (rGO)-polyaniline (PANI) supercapacitors with LiCl, Li2SO4, and H2SO4 electrolytes. *J. Electrochem. Soc.* **2023**, *170* (1), 010532.
(15) Xie, R.-J.; Cheng, I.-C.; Chen, J.-Z. East asian calligraphy black ink-coated paper as flexible conducting electrode for supercapacitor. *ECS J. Solid State Sci. Technol.* **2021**, *10* (12), 123013.
(16) Ben, J.; Song, Z.; Liu, X.; Lü, W.; Li, X. Fabrication and electrochemical performance of PVA/CNT/PANI flexible films as electrodes for supercapacitors. *Nanoscale Res. Lett.* **2020**, *15* (1), 151.
(17) Liu, C.; Hung, C.-W.; Cheng, I.-C.; Hsu, C.-C.; Cheng, I.-C.; Chen, J.-Z. Dielectric barrier discharge plasma jet (DBDjet) processed reduced graphene oxide/polypyrrole/chitosan nanocomposite supercapacitors. *Polym.* **2021**, *13* (20), 3585.
(18) Chen, C.-S.; Ni, I.-C.; Wu, C.-I.; Hsu, C.-C.; Cheng, I.-C.; Chen, J.-Z. Flexible asymmetric supercapacitors with reduced graphene oxide and lithium manganese oxide electrodes processed by atmospheric-pressure plasma jet. *Ceram. Int.* **2025**, *51* (16), 22849–22859.
(19) Chen, L.-H.; Ni, I.-C.; Wu, C.-I.; Yu, S.-E.; Hsu, C.-C.; Cheng, I.-C.; Chen, J.-Z. Flexible asymmetric supercapacitors based on zinc cobalt composites on stainless steel Fiber paper processed by atmospheric-pressure plasma jet. *J. Electroanal. Chem.* **2025**, 119411.
